# Juvenile idiopathic arthritis and rheumatoid arthritis: a narrative review of age-dependent, subtype-specific immune mechanisms, biomarkers, and treatment strategies

**DOI:** 10.3389/fimmu.2026.1873473

**Published:** 2026-10-27

**Authors:** Wenwen Sheng, Xinyue Yang, Zhenzhe Yu, Yongqi Yang, Huihe Chen, Yang Cheng, Weijie Wang

**Affiliations:** 1 The Second Clinical Medical College of Zhejiang Chinese Medical University, Hangzhou, China; 2 State Key Clinical Specialty of Rheumatology, Department of Rheumatology, the Second Affiliated Hospital of Zhejiang Chinese Medical University, Hangzhou, China

**Keywords:** juvenile idiopathic arthritis, rheumatoid arthritis, developmental immunology, disease endotypes, biomarkers, treatment strategies, macrophage activation syndrome

## Abstract

**Background:**

Juvenile idiopathic arthritis (JIA) and adult rheumatoid arthritis (RA) share chronic inflammatory arthritis, but their biological drivers and implications vary by developmental stage and phenotype. JIA arises during immune and skeletal maturation and encompasses heterogeneous conditions, whereas adult RA develops after skeletal maturity. Existing reviews have considered the diseases separately or focused on polyarticular JIA, leaving uncertain which adult RA-derived mechanisms, biomarkers, and treatment principles transfer to JIA phenotypes.

**Main body:**

A targeted search of PubMed/MEDLINE and Web of Science through 25 September 2026 informed an age-by-endotype framework comparing JIA phenotypes with adult RA across immune and tissue mechanisms, structural consequences, biomarker function and evidence maturity, and treatment mechanism, exposure, and safety. Systemic JIA, conceptualized within the Still’s disease spectrum, diverges most from adult RA because of myeloid-cell activation, macrophage activation syndrome risk, and inflammation associated with interleukin-1, interleukin-6, and interleukin-18. Rheumatoid factor (RF)-positive polyarticular JIA shows partial convergence with adult seropositive RA through autoantibody-associated adaptive immunity, chronic synovitis, and erosive potential, but development and pediatric safety preclude equivalence. Oligoarticular JIA is distinguished by synovitis and uveitis, whereas enthesitis-related arthritis and juvenile psoriatic arthritis align more closely with entheseal and psoriatic-spectrum biology than with RA. Biomarker interpretation and treatment are phenotype-specific: ferritin, interleukin-18, and S100 proteins inform systemic hyperinflammation; RF and anti-citrullinated protein antibodies characterize RA-like polyarthritis; antinuclear antibodies inform uveitis surveillance; and human leukocyte antigen B27 supports classification in enthesitis-related arthritis. Treatment ranges from early interleukin-1 or interleukin-6 inhibition in systemic JIA to methotrexate, biologic disease-modifying antirheumatic drugs, and selected Janus kinase inhibitor use in RA-like polyarthritis after consideration of regulatory approval and individualized risk, and therapy directed at tumor necrosis factor or interleukin-17 in entheseal or psoriatic disease. Tissue, imaging, multi-omic, microbiome, and treatment-response biomarkers remain investigational.

**Conclusions:**

The relationship between JIA and adult RA is phenotype- and domain-specific rather than universal. This framework identifies where adult-derived evidence can inform pediatric interpretation and where direct pediatric validation is required. Longitudinal, subtype-defined pediatric cohorts integrating tissue, imaging, and multi-omic data are needed to support mechanism-based, developmentally appropriate care.

## 1 Introduction

Juvenile idiopathic arthritis (JIA) is an umbrella term for a heterogeneous group of inflammatory arthritides of unknown origin that begin before the age of 16 years and persist for more than 6 weeks after alternative causes have been excluded (1). Rheumatoid arthritis (RA), by contrast, is a chronic inflammatory autoimmune disease that primarily affects synovial joints and is frequently associated with autoantibody production, progressive structural damage, and disability (2). Both conditions can result in persistent joint inflammation and long-term functional impairment, while RA continues to impose a substantial global burden, with the total number of affected individuals projected to increase markedly by 2050 (1–3). Nevertheless, their shared presentation as chronic inflammatory arthritis does not mean that JIA represents a childhood-onset form of adult RA.

JIA is not a single biological entity, and its marked subtype heterogeneity is central to comparisons with adult RA. Among the JIA subtypes, rheumatoid factor (RF)-positive polyarticular JIA shares several clinical features with adult seropositive RA, but this resemblance does not imply biological equivalence. In patients with polyarticular-course JIA persisting into adulthood, RF and anti-citrullinated protein antibody (ACPA) positivity are associated with greater structural joint damage, supporting the prognostic relevance of autoantibodies in this RA-like pediatric phenotype (1, 4). Systemic JIA, increasingly conceptualized within the age-spanning Still’s disease spectrum, is distinguished by systemic inflammation and the risk of macrophage activation syndrome (5, 6). Oligoarticular JIA is defined by limited joint involvement, while JIA-associated uveitis remains an important complication; younger age at onset and antinuclear antibody (ANA) positivity were identified as major risk factors in a recent meta-analysis (1, 7). Enthesitis-related arthritis belongs to the juvenile spondyloarthritis spectrum and is characterized by enthesitis and peripheral arthritis, with sacroiliac or axial involvement occurring in some patients (8).

Age represents a second major dimension of disease heterogeneity. JIA develops during ongoing linear growth and pubertal maturation, and chronic inflammation and glucocorticoid exposure may adversely affect growth and skeletal development (9). In adult RA, persistent synovial inflammation is associated with progressive joint damage and functional disability (2). These differences indicate that the developmental context in which inflammation occurs should be considered when interpreting tissue injury, disease progression, biomarkers, and therapeutic requirements in childhood and adult inflammatory arthritis.

Recent tissue-based studies have provided direct evidence that JIA synovitis cannot be understood solely through conventional clinical categories or by assuming equivalence with adult RA. A 2025 synovial tissue atlas identified distinct immune and stromal niches associated with JIA severity and demonstrated age-associated differences in cellular composition between pediatric JIA and adult RA synovium (10). High-resolution spatial transcriptomic analysis subsequently revealed spatially organized interactions among fibroblasts, endothelial cells, macrophages, T cells, and other cellular populations within inflamed JIA synovium (11). These findings provide a tissue-level framework for understanding pediatric synovitis and complement emerging biomarker research. However, most candidate biomarkers still require validation in well-defined, subtype-specific pediatric cohorts before routine clinical application (12).

Major reviews describe JIA as an umbrella diagnosis encompassing clinically distinct categories with phenotype-specific manifestations and management considerations (1, 13). A recent biomarker-focused review separately summarizes candidate markers for diagnosis, prognosis, disease monitoring, and treatment selection in JIA (12). A major RA review synthesizes autoantibody-associated autoimmunity, synovial inflammation, progressive joint damage, and therapeutic strategies directed at remission or low disease activity in adults (2). Cross-age literature has further examined how age-related mechanisms shape immune-mediated arthritis (14), how genetic evidence relates pediatric and adult arthritis classification (15), and how polyarticular JIA compares with adult RA in pathogenesis, clinical manifestations, and treatment options (16). A biological-classification review has further proposed age-spanning mechanistic groupings based on antibody dependence, synovitis versus enthesitis, and autoimmune versus autoinflammatory biology (17). Collectively, these reviews establish subtype heterogeneity within JIA, the major immunopathological framework of adult RA, and several age-spanning biological relationships. However, direct JIA-RA comparisons have concentrated mainly on polyarticular disease and have not evaluated the transferability of adult RA-derived evidence across multiple representative JIA phenotypes and clinical domains.

The key unresolved question is therefore not whether JIA and adult RA are globally similar or dissimilar, but which adult RA-derived mechanistic insights, biomarker evidence, and treatment principles are transferable to individual JIA phenotypes and under what developmental constraints. A direct comparison is necessary because chronic inflammatory arthritis can arise from distinct immune programs and tissue compartments, including the synovium and enthesis (1, 17). JIA and its treatment can also affect skeletal growth and pubertal development (9), whereas age-dependent immune maturation and the cumulative pro-inflammatory burden of environmental exposures influence arthritis emergence and heterogeneity (14). Without separating developmental effects from subtype-specific biology, similarities may therefore be overgeneralized, while legitimate opportunities for cross-age translation may also be missed.

To address this gap, we introduce an age-by-endotype transferability framework, a qualitative conceptual model that positions each JIA phenotype along two dimensions: endotypic proximity to adult RA and developmental compatibility with adult-derived evidence (**Figure 1**). Transferability is evaluated separately across four domains: (i) dominant immune pathway and tissue compartment; (ii) structural consequences of inflammation; (iii) biomarker function and evidence maturity; and (iv) treatment mechanism, exposure, and safety. Within each domain, transferability is classified as High, Conditional, or Low according to the directness and consistency of pediatric evidence, developmental compatibility, phenotype-specific complications, treatment exposure and safety, and the degree of mechanistic concordance with adult RA. RF-positive polyarticular JIA shows the clearest endotypic proximity to adult seropositive RA on the basis of genetic evidence (18). In the broader polyarticular JIA trial population, exposure-matched analyses have also demonstrated response similarity to adult RA for several biologic agents (19). This combination supports High mechanistic but only Conditional treatment-level transferability, because the response evidence is not specific to the RF-positive subgroup and does not establish complete biological equivalence. Systemic JIA, by contrast, lies toward the autoinflammatory end of the Still’s disease spectrum but should not be regarded as a purely innate immune disease; phase-dependent immune mechanisms and phenotype-specific complications such as macrophage activation syndrome make transferability from adult RA restricted and domain-specific (6, 20, 21). Other JIA phenotypes should likewise be assessed separately because biologically relevant distinctions—including synovitis versus enthesitis and antibody-associated versus antibody-independent disease—may alter the applicability of adult RA evidence (17). The framework is intended as an organizing synthesis for this review rather than as a validated classification or scoring system.

**Figure 1:**
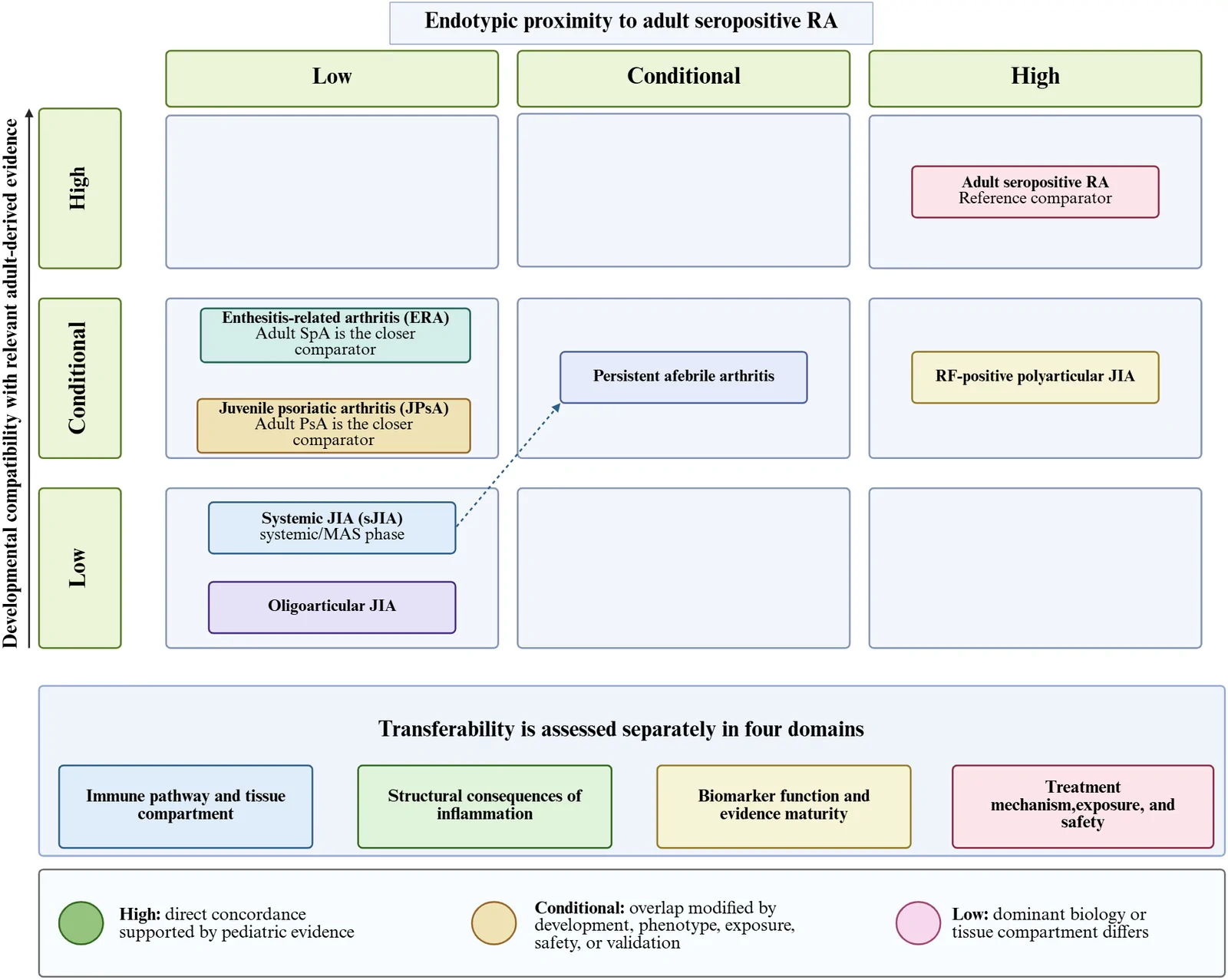
Age-by-endotype transferability framework across representative juvenile idiopathic arthritis phenotypes and adult seropositive rheumatoid arthritis. JIA phenotypes are positioned according to their endotypic proximity to adult seropositive RA and the developmental compatibility of relevant adult-derived evidence. Endotypic proximity was judged qualitatively from dominant immune pathways, tissue compartments, autoantibody profiles, and structural disease patterns, whereas developmental compatibility was judged from the availability of direct pediatric evidence and the extent to which growth, maturation, dosing, treatment exposure, and long-term safety modify adult-derived evidence. High, conditional, and low indicate broad concordance, partial or context-dependent overlap, and substantial divergence, respectively. The dashed arrow indicates a potential transition, in a subset of patients, from the systemic/MAS phase of systemic JIA to persistent afebrile arthritis. Adult SpA and adult PsA are shown as more appropriate phenotype-specific comparators for ERA and JPsA, respectively. The positions represent an author-derived qualitative synthesis of the reviewed evidence and do not constitute a validated classification or scoring system.

## 2 Methodology

This narrative review used a structured, targeted search of PubMed/MEDLINE and Web of Science covering 1 January 2015 to 25 September 2026. Searches were conducted in two stages: a direct comparative search combining terms for JIA and adult RA, followed by phenotype- and domain-specific searches addressing systemic JIA, RF-positive polyarticular JIA, oligoarticular JIA, enthesitis-related arthritis, juvenile psoriatic arthritis, developmental biology, synovial tissue, biomarkers, and treatment. Controlled vocabulary, where available, was combined with free-text terms, and the complete database-specific search strategies are provided in **Supplementary Table S1**. Reference lists of eligible studies, relevant reviews, classification criteria, consensus statements, and treatment recommendations were screened manually to identify additional publications. Searches were limited to English-language publications. Recent evidence was prioritized, whereas earlier landmark studies were retained when they established classification systems, foundational mechanisms, validated outcome measures, or treatment principles.

Eligible sources included peer-reviewed primary studies, systematic reviews and meta-analyses, published classification criteria, and evidence-based guidelines or official consensus recommendations. Human tissue, ex vivo, and animal studies were eligible when they directly informed a predefined mechanistic or tissue-level comparison. Sources were included if they addressed at least one representative JIA phenotype or adult RA and provided evidence relevant to developmental or structural pathology, immune mechanisms, biomarkers, treatment efficacy, or safety. Sources concerning adult spondyloarthritis or psoriatic arthritis were included only when needed to contextualize enthesitis-related arthritis or juvenile psoriatic arthritis domains that lack an appropriate adult RA counterpart. Duplicate reports, non-English publications, studies outside the predefined phenotypes or comparison domains, and reports lacking sufficient methodological detail to evaluate the cited claim were excluded. After deduplication, one reviewer screened titles and abstracts and assessed potentially eligible full texts; a second reviewer checked the final inclusion decisions, and disagreements were resolved through discussion. The searches identified 8,716 database records and 47 additional records through reference-list screening and supplementary sources. After 2,777 duplicates were removed, 5,986 records remained for title-and-abstract screening. Because full-text assessment was not recorded as a distinct PRISMA-style screening stage in this narrative review, the number of full-text reports assessed was not separately documented. The final narrative synthesis included 117 unique publications.

To distinguish the evidentiary basis of individual claims, included studies were characterized by design as systematic reviews or meta-analyses, randomized controlled trials, longitudinal cohorts, cross-sectional or retrospective studies, single-cell or spatial transcriptomic studies, and animal, ex vivo, or other mechanistic studies. The strength of evidence was appraised qualitatively at the claim level rather than assigned uniformly to an entire publication. Four descriptive categories were used: Established, Supportive, Limited, and Exploratory. Established evidence comprised consistent, directly applicable, and independently replicated findings from multiple robust human studies or systematic syntheses of sufficiently homogeneous evidence. Supportive evidence included a single randomized trial, a well-designed prospective cohort, or concordant findings from multiple human studies that lacked sufficient replication or generalizability. Limited evidence included findings derived primarily from small, retrospective, cross-sectional, single-center, *post hoc*, or phenotypically heterogeneous studies. Exploratory evidence comprised discovery-stage single-cell, spatial transcriptomic, multi-omic, ex vivo, or animal findings without independent clinical validation. Study design alone did not determine the evidence category; directness to the specified JIA phenotype and age group, sample size, outcome validity, replication, consistency, and clinical validation were also considered. When findings conflicted, greater weight was given to direct phenotype-specific pediatric evidence, prospective or controlled designs, larger samples, validated outcomes, and independent replication; unresolved inconsistencies were reported explicitly rather than resolved by counting the number of supporting publications. Systematic reviews were not automatically categorized as Established when their underlying evidence was heterogeneous or methodologically limited. Guidelines were used to describe current recommendations, but recommendation strength and certainty of the supporting evidence were considered separately; a strong recommendation did not automatically constitute Established evidence. Mechanistic findings were not interpreted as evidence of diagnostic, prognostic, predictive, or treatment-selection utility without appropriate clinical validation. The wording of conclusions was calibrated to these categories, which were used as an organizing framework rather than a validated scoring system. Because this was a narrative review, no formal meta-analysis, protocol registration, or study-level risk-of-bias assessment was performed.

## 3 Comparative clinical phenotypes of representative JIA subtypes and adult RA

JIA and adult RA share chronic inflammatory arthritis as a clinical endpoint (1, 2), but the biological significance of this overlap varies substantially across JIA phenotypes (1). RF-positive polyarticular JIA shows the closest phenotypic and genetic relationship to adult autoantibody-positive RA (18), while RF and ACPA positivity were associated with greater radiographic structural damage among patients with polyarticular-course JIA persisting into adulthood (4). These convergences should not be interpreted as evidence of complete biological equivalence. Other JIA phenotypes are distinguished by different clinical patterns: systemic JIA by systemic hyperinflammation and the risk of macrophage activation syndrome (5); oligoarticular JIA by its association with uveitis (7, 22); enthesitis-related arthritis by enthesitis and possible axial involvement (8); and juvenile psoriatic arthritis by psoriasis-associated manifestations such as dactylitis and nail abnormalities (23). Together, these contrasts show that adult RA is a close comparator only for selected JIA phenotypes, particularly RF-positive polyarticular JIA, whereas systemic, oligoarticular, entheseal, and psoriatic phenotypes require distinct biological and clinical interpretation (**Table 1**).

**Table 1:** Phenotype-specific clinical anchors across representative juvenile idiopathic arthritis subtypes and adult rheumatoid arthritis.

Disease category	Articular phenotype	Systemic or extra-articular phenotype	Comparative clinical anchors	References
Systemic JIA (within the Still’s disease spectrum)	Arthritis may accompany or follow the systemic inflammatory manifestations. Joint distribution is variable, and persistent polyarthritis may develop.	Quotidian fever and an evanescent erythematous rash are characteristic. Generalized lymphadenopathy, hepatomegaly and/or splenomegaly, and serositis may occur. Macrophage activation syndrome (MAS) is a major potentially life-threatening complication.	Onset/context: Before age 16 years, commonly in early childhood. Systemic JIA shares an age-spanning cytokine-driven disease framework with adult-onset Still’s disease, while retaining pediatric-specific developmental, dosing, safety, MAS, and lung-disease considerations. Classification/routine laboratory anchors: Neutrophilic leukocytosis, anemia, thrombocytosis, elevated ESR and CRP, and often hyperferritinemia; RF and ANA are usually negative. Course/structural risk: The disease course may be monophasic, polycyclic, or persistent. Systemic inflammation and MAS dominate the early risk, whereas persistent arthritis may contribute to later joint damage.	(5, 6, 53, 55, 69, 89, 102–104)
RF-positive polyarticular JIA	Arthritis affects five or more joints during the first 6 months of disease. The pattern is usually symmetric and commonly involves the small joints, although large joints may also be affected.	No subtype-defining systemic or extra-articular manifestation.	Onset/context: Usually begins in late childhood or adolescence, with marked female predominance. Classification/routine laboratory anchors: IgM RF is positive on at least two occasions at least 3 months apart. ACPA is frequently detected but is not part of the ILAR classification criteria; ESR and CRP may be elevated. Course/structural risk: This phenotype shows the closest clinical and genetic relationship to adult autoantibody-positive RA. Drug-free remission is uncommon, and RF and ACPA positivity are associated with increased structural-damage risk, particularly when polyarticular disease persists into adulthood.	(4, 18, 69, 105, 106)
Oligoarticular JIA	Arthritis affects one to four joints during the first 6 months of disease. Joint involvement is usually asymmetric and predominantly affects large joints, particularly the knees and ankles.	Chronic anterior uveitis is an important complication and is frequently asymptomatic at onset.	Onset/context: Usually begins in early childhood, with female predominance. Classification/routine laboratory anchors: ANA is frequently positive; ESR and CRP may be normal or mildly elevated. Young age at disease onset and ANA positivity are the most consistent independent predictors of uveitis. Course/structural risk: Disease may remain limited to four or fewer joints or extend to involve more than four joints after the first 6 months. Disease extension and uveitis may adversely affect long-term outcome.	(7, 22, 69, 105–109)
Enthesitis-related arthritis	Arthritis and enthesitis are the principal musculoskeletal manifestations. Peripheral arthritis commonly affects the lower limbs, while sacroiliac or axial involvement may occur.	Acute anterior uveitis may occur.	Onset/context: Usually begins in late childhood or adolescence and is more common in boys. Male sex with disease onset after the sixth birthday is an ILAR classification feature. Classification/routine laboratory anchors: HLA-B27 is frequently positive; repeated IgM RF positivity is an ILAR exclusion criterion. ESR and CRP may be elevated but are nonspecific. Course/structural risk: Sacroiliac or axial disease may become apparent later. Hip or ankle involvement and restricted spinal mobility are associated with poorer outcome. HLA-B27 positivity has been associated with a lower probability of remission, although this relationship may partly reflect accompanying sacroiliitis, enthesitis, or hip involvement.	(8, 69, 110–112)
Juvenile psoriatic arthritis	Arthritis is often oligoarticular at onset but may extend to a polyarticular course. Knees, ankles, wrists, and small joints may be involved. Distal interphalangeal joint disease, dactylitis, enthesitis, or axial involvement may occur.	Psoriasis and nail abnormalities, including nail pitting and onycholysis, are characteristic. Chronic anterior uveitis may occur, particularly in younger ANA-positive patients.	Onset/context: A bimodal presentation has been described. The early-onset group is often female and ANA-positive, whereas the later-onset group more commonly exhibits enthesitis and other spondyloarthritis-like features. Classification/routine laboratory anchors: Under ILAR criteria, JPsA is defined by arthritis with psoriasis or, in the absence of psoriasis, arthritis with at least two of the following: dactylitis, nail pitting or onycholysis, and psoriasis in a first-degree relative. Course/structural risk: The phenotype is heterogeneous. Psoriasis may develop years after arthritis, oligoarthritis may extend, and radiographic joint damage can occur.	(23, 69)
Adult rheumatoid arthritis	Persistent inflammatory polyarthritis commonly presents with symmetric involvement of the hands, wrists, and other small joints.	Rheumatoid nodules and pulmonary disease, including interstitial lung disease, are recognized extra-articular manifestations.	Onset/context: Occurs in adulthood after skeletal maturation. Classification/routine laboratory anchors: RF and/or ACPA support classification; ACPA is generally more specific than RF, while ESR and CRP reflect inflammatory activity. Course/structural risk: Persistent synovitis may lead to bone erosion, cartilage loss, deformity, disability, and systemic complications. RF or ACPA positivity and structural damage at presentation indicate a greater risk of further progression.	(2, 68, 113–117)

ACPA, anti-citrullinated protein antibodies; ANA, antinuclear antibodies; CRP, C-reactive protein; ESR, erythrocyte sedimentation rate; HLA-B27, human leukocyte antigen B27; IgM, immunoglobulin M; ILAR, International League of Associations for Rheumatology; JIA, juvenile idiopathic arthritis; JPsA, juvenile psoriatic arthritis; MAS, macrophage activation syndrome; RA, rheumatoid arthritis; RF, rheumatoid factor.

## 4 Age-dependent pathophysiological differences between JIA and RA

Because JIA develops during ongoing bone modeling and acquisition of peak bone mass, chronic inflammation, reduced physical activity, nutritional disturbance, and glucocorticoid exposure may impair bone mineral accrual (9). By contrast, adult RA affects a skeletally mature host, in whom persistent synovitis is associated with focal erosions, periarticular bone loss, and systemic osteoporosis (24). In a cross-sectional study of 60 Egyptian children with JIA, 31% had a lumbar spine BMD Z score below −2, and multivariable analysis associated decreased BMD with disease duration exceeding 36 months and glucocorticoid use (25). This pediatric prevalence estimate is limited by the study’s cross-sectional design, small sample, subtype composition, and setting. Accordingly, adult RA evidence on inflammation-mediated bone loss is conditionally transferable to JIA, but bone-mineral outcomes in children must also be interpreted in the context of ongoing bone-mass accrual.

In JIA, persistent synovitis is also organized through interactions between immune and stromal cells, but direct pediatric tissue evidence remains emerging. A multimodal analysis of synovial biopsies from 19 treatment-naïve children identified spatial niches containing macrophage, lymphocyte, endothelial, and stromal populations; comparison with adult-onset inflammatory arthritis revealed both shared pathogenic cell populations and age-associated differences in tissue vascularity, the prominence of innate immunity, and TGF-β-responsive stromal subsets (10). A separate spatial transcriptomic study of synovial tissue from nine patients with active JIA identified endothelial–fibroblast and macrophage–T-cell interactions and reported JIA-enriched NOTCH3+ and CXCL12+ sublining fibroblast states relative to RA (11). In adult RA, by comparison, extensive mechanistic evidence supports a self-sustaining synovial circuit in which infiltrating immune cells and activated fibroblast-like synoviocytes amplify inflammation, while immune–fibroblast signaling promotes RANKL-dependent osteoclast activation (24, 26, 27). These findings support partial overlap in immune–stromal organization, but the pediatric evidence remains Exploratory because it derives from small tissue cohorts and has not established the stability, prognostic significance, or treatment responsiveness of the identified cell states. Accordingly, adult RA concepts of immune–stromal persistence and osteoclast coupling have conditional, rather than complete, transferability to JIA; specific pediatric fibroblast states and spatial niches require independent validation.

Longitudinal growth occurs through chondrocyte proliferation and hypertrophy at the growth plate and subsequent endochondral ossification (9). In JIA, chronic inflammation may disrupt this process through systemic effects on the GH–IGF-1 axis and local cytokine effects on growth-plate chondrocytes; undernutrition, reduced physical activity, delayed puberty, and glucocorticoid exposure can further modify growth (9). Localized inflammation can also produce asymmetric growth. When one lower limb is predominantly affected, leg-length discrepancy may occur across monoarticular, oligoarticular, and polyarticular disease patterns; local bone growth may be accelerated or inhibited depending on the site of inflammation and age at disease onset (28). Adult RA also involves inflammation-mediated disruption of bone remodeling (24). However, because adult RA occurs after skeletal maturation, it has no direct counterpart to growth-plate dysfunction or altered longitudinal growth. Longitudinal clinical evidence indicates that growth impairment is neither inevitable nor uniform across JIA. In the 568-patient UK Childhood Arthritis Prospective Study inception cohort, 39% met the study-defined criterion for growth restriction—a decrease in height Z score of at least 0.5 over three years—although median height remained within the population range (29). In the prospective multicenter ReACCh-Out cohort of 1,147 children, mean height Z scores and the prevalence of short stature remained stable overall, whereas systemic JIA, uncontrolled disease activity, and prolonged systemic glucocorticoid exposure were associated with greater risk of growth impairment (30). These longitudinal observational cohorts provide Supportive evidence, but differences in growth definitions, cohort composition, treatment exposure, and analytic approaches preclude a single risk estimate applicable to all JIA phenotypes (29, 30). Accordingly, adult RA evidence on inflammation-mediated bone remodeling is conditionally transferable to JIA, whereas linear-growth and growth-plate outcomes require pediatric, age- and phenotype-specific longitudinal evidence.

Musculoskeletal impairment in JIA extends beyond the inflamed joints. In a small cross-sectional study of 32 children and adolescents with predominantly low disease activity and 18 matched controls, patients with JIA reported greater pain and fatigue and showed lower physical activity, walking speed, and six-minute walk distance, although quadriceps strength did not differ between groups (31). In a separate controlled cross-sectional study of 60 patients aged 10–16 years and 60 matched controls, JIA was associated with lower muscle strength and total-body bone mineral density, despite comparable cardiorespiratory fitness and body composition (32). A recent scoping review of 37 studies involving 2,040 patients found heterogeneous evidence for reduced muscle mass but more consistent evidence for reduced muscle strength, with deficits related to disease activity and disability (33). Adult RA similarly involves loss of skeletal muscle strength and mass; chronic inflammation disrupts muscle homeostasis, and these deficits contribute to physical disability in addition to joint inflammation and damage (34). Thus, muscle weakness represents a shared musculoskeletal consequence across age groups, but the contribution of growth, physical development, disease phenotype, and treatment exposure remains specific to JIA. Intervention evidence remains Limited: a single 12-week randomized trial involving 48 adolescents with polyarticular JIA found that lower-body aqua-plyometric training improved quadriceps peak torque, bone-mineral measures, and physical fitness relative to standard exercise (35). Overall, the pediatric evidence is Supportive for impaired muscle strength but Limited for muscle-mass loss and exercise efficacy; adult RA findings are therefore conditionally transferable rather than directly generalizable to JIA.

The temporomandibular joint represents a developmentally distinctive site of involvement in JIA because inflammatory or mechanical damage to the condylar cartilage before skeletal maturity may disrupt mandibular growth (36). JIA-related TMJ arthritis can consequently cause orofacial symptoms, orofacial dysfunction, and dentofacial deformity (37). Evidence concerning established deformity remains Limited: a systematic review of surgical correction included 28 publications, of which 24 were case reports or case series, indicating that its findings primarily describe selected patients with severe disease rather than the risk among all patients with TMJ involvement (38). In a longitudinal MRI study of 38 children receiving systemic treatment, TMJ inflammation improved over a median of 3.6 years, osseous deformity improved in joints with arthritis at baseline, and mandibular ramus growth did not differ significantly between joints with and without baseline inflammation; however, the observational design does not establish a causal treatment effect (39). Adult RA can also involve the TMJ, and a systematic review and meta-analysis found that RA was associated with a higher occurrence of CBCT-detected osseous changes, including erosion, flattening, sclerosis, and osteophytes (40). Thus, inflammation-related structural damage of the TMJ is shared across age groups, whereas altered mandibular and facial growth is specific to disease occurring before skeletal maturity. Adult RA evidence is conditionally transferable for structural TMJ damage but has low transferability for growth-related outcomes, which require pediatric longitudinal evidence.

Taken together, adult RA evidence has conditional transferability to shared mechanisms of synovitis, bone erosion, muscle weakness, and structural TMJ damage, whereas growth-plate injury, craniofacial growth disturbance, and pediatric exercise responses require age-specific evidence (**Figure 1**).

## 5 Age-dependent etiological pathways in JIA and RA

The etiologies of both JIA and adult RA are multifactorial, but the relevant pathways and strength of evidence differ across phenotypes and age groups. A multi-omics review describes JIA as clinically and biologically heterogeneous: a small proportion of cases are attributable to monogenic causes, whereas most involve polygenic susceptibility together with subtype-specific genetic, epigenetic, and transcriptomic patterns (41). In adult RA, contemporary etiological models likewise integrate genetic susceptibility, environmental exposures, mucosal immune dysregulation, and joint-tissue mechanisms (42). These reviews provide supportive evidence for multifactorial causation in both diseases, but they do not establish a single shared causal pathway or direct equivalence between individual JIA phenotypes and adult RA. Accordingly, adult RA etiological models have conditional transferability to JIA and should be evaluated separately for each phenotype and developmental context.

Genetic susceptibility contributes to both JIA and adult RA, but its architecture varies across JIA phenotypes. Reviews of JIA genetics identify HLA and non-HLA immune-regulatory loci and describe partially overlapping, subtype-specific genetic patterns (41, 43). A comparative analysis of genome-wide association study summary statistics found extensive overlap between JIA and RA in the HLA region, several shared non-HLA variants, and enrichment of 32 shared pathways; however, the JIA dataset pooled clinical subtypes and was substantially smaller than the RA dataset, limiting phenotype-specific inference (44). Subtype-focused evidence indicates that RF-positive polyarticular JIA is genetically closest to adult seropositive RA, whereas oligoarticular and RF-negative polyarticular JIA have overlapping but nonidentical immunogenetic profiles (43). Systemic JIA, within the Still’s disease spectrum, has HLA and non-HLA susceptibility signals in a mixed autoinflammatory and adaptive immune context (20), while enthesitis-related arthritis has a distinct HLA-B27-associated background (8). Genetic evidence specific to juvenile psoriatic arthritis remains Limited (23). Overall, the evidence is Supportive for shared genetic susceptibility but insufficient to establish a single genetic architecture across JIA and adult RA. Adult seropositive RA genetic evidence therefore has high transferability to RF-positive polyarticular JIA, conditional transferability to oligoarticular and RF-negative polyarticular JIA, low transferability to systemic JIA and enthesitis-related arthritis, and currently uncertain transferability to juvenile psoriatic arthritis.

Beyond genetic susceptibility, environmental exposures have been investigated as potential modifiers of JIA risk, particularly during prenatal and early-life development. A 2022 systematic review of 39 observational studies examined 66 environmental factors, but only eight factors from ten studies could be meta-analyzed; cesarean delivery was associated with higher JIA risk, whereas having siblings and maternal prenatal smoking showed inverse associations (45). A later systematic review of 23 observational studies also reported a positive association with cesarean delivery and inverse associations with heavy maternal or gestational smoking, while emphasizing limited comparability among studies (46). These heterogeneous and partly counterintuitive observational findings do not establish causal or protective effects and provide limited evidence for any single exposure across JIA phenotypes. In adult RA, long-duration cigarette smoking and other respiratory exposures, obesity, and dietary patterns have been more consistently linked to disease risk, including interactions with genetic susceptibility (42). Accordingly, the environmental evidence is Limited for JIA and Supportive for adult RA, and adult RA exposure models have only conditional, phenotype-specific transferability to JIA.

Microbial signals may link environmental exposures to immune regulation, but pediatric evidence remains predominantly associative (47, 48). A 2025 review summarized reported differences in the oral and fecal microbiota of patients with JIA and discussed their implications for future research (47). A 2026 review noted that gut–joint research in JIA remains less developed than that in adult RA and experimental arthritis; proposed mechanisms include dysbiosis, impaired epithelial barrier function, altered microbial metabolites, and trafficking of gut-recirculating immune cells, but these mechanisms are derived largely from experimental and adult studies and remain insufficiently tested across JIA phenotypes (48). In a small cross-sectional study, 10 HLA-B27-positive and 5 HLA-B27-negative patients with JIA-ERA were compared with 10 healthy controls. HLA-B27-positive patients had higher fecal calprotectin levels than both comparison groups and showed differences in the abundance of several bacterial taxa; alpha and beta diversity were similar between the two JIA-ERA groups but differed from healthy controls (49). The small sample and cross-sectional design preclude causal inference and limit generalization beyond ERA. Overall, microbiome evidence in JIA is classified as Limited, whereas the HLA-B27-stratified ERA findings are Exploratory. Adult RA gut–joint evidence therefore has only conditional transferability to JIA, with the clearest current rationale in ERA.

Infections have been investigated as possible contributors to JIA onset, but the evidence does not identify a common causal pathogen. A 2022 systematic review reported positive associations for all-cause infections and antibiotic exposure in some observational studies, but found little consistent evidence for specific pathogens (45). An earlier etiological review considered parvovirus B19, Epstein–Barr virus, and several bacterial species, yet concluded that the available evidence neither established nor excluded causal associations (50). Infection should therefore be described as a possible trigger in genetically susceptible children rather than as an established cause of persistent JIA. In adult RA, viral and other respiratory infections have also been associated with disease risk, but current etiological models place them among multiple interacting respiratory, mucosal, metabolic, and genetic factors (42). Overall, infection-related evidence in JIA is Limited, and adult RA infection-related models have only conditional, phenotype-specific transferability to JIA.

In adult seropositive RA, a multistage preclinical process has been proposed in which genetic susceptibility and cumulative environmental exposures precede clinically apparent synovitis. Current mucosal-origin models implicate site-specific processes in the lung, oral-periodontal tissues, and gut, including local inflammation, dysbiosis, and, in some individuals, local production of RA-related autoantibodies; however, mucosal involvement is heterogeneous, and no single site or pathway accounts for all cases (42, 51). These processes may contribute to loss of immune tolerance and systemic expansion of RF and ACPA responses before joint inflammation becomes clinically evident (42, 51). In a prospective cohort of 82 IgG ACPA-positive individuals with musculoskeletal pain but no clinical arthritis at baseline, 48% developed at least one arthritic joint during a mean follow-up of six years, and baseline levels of secretory component-containing ACPA independently predicted progression (52). This study provides Supportive rather than Established evidence because it was a single, modest-sized at-risk cohort and does not establish the anatomical site in which these antibodies originated. Accordingly, the adult RA mucosal model has conditional transferability to RF-positive polyarticular JIA but low transferability to systemic JIA, oligoarticular JIA, enthesitis-related arthritis, and juvenile psoriatic arthritis.

Taken together, etiological overlap between JIA and adult RA is phenotype-specific rather than global. Genetic and autoimmune susceptibility provides the strongest cross-age bridge between RF-positive polyarticular JIA and adult seropositive RA, whereas systemic JIA and enthesitis-related arthritis follow more distinct autoinflammatory and HLA-B27-associated trajectories, respectively. In JIA, early-life exposures act during immune maturation and microbiome development, whereas adult seropositive RA more often reflects cumulative environmental and mucosal exposures operating across a longer preclinical interval. Evidence for individual environmental, infectious, and microbiome-related triggers in JIA remains predominantly Limited or Exploratory. Adult RA etiological models are therefore conditionally transferable only when developmental context, disease phenotype, and the implicated biological pathway are concordant. These etiological differences are reflected in distinct immune-cell and cytokine programs across JIA phenotypes.

## 6 Subtype-specific immune pathways: immune-cell circuits and targetable inflammatory axes

### 6.1 Systemic JIA: myeloid-cell activation and cytokine amplification

Systemic JIA is increasingly conceptualized together with adult-onset Still’s disease within a unified Still’s disease spectrum rather than as an RA-like disorder (6). This age-spanning framework recognizes shared cytokine-driven systemic inflammation while preserving pediatric-specific considerations, including immune and skeletal development, macrophage activation syndrome surveillance, systemic JIA-associated lung disease, dosing, and long-term safety. Within the JIA spectrum, systemic JIA lies toward the autoinflammatory end but should not be regarded as a purely innate immune disease. Its early systemic phase is dominated by innate immune activation and cytokine amplification, with prominent involvement of myeloid cells and IL-1, IL-6, and IL-18 pathways; IFN-γ is particularly relevant to MAS-associated hyperinflammation (5, 20). In a subset of patients, systemic inflammation may evolve into persistent afebrile arthritis with greater participation of adaptive immune mechanisms, but this proposed biphasic model is not universal and requires further validation (21). Adult seropositive RA, by contrast, is principally organized around autoantibody-associated adaptive immunity and persistent synovial immune–stromal interactions (2, 24). Evidence for innate cytokine dominance in the early systemic phase of systemic JIA is Established, whereas evidence for a transition toward adaptive immune-driven arthritis is Supportive.

Ferritin and S100 proteins, particularly S100A8/A9 (calprotectin) and S100A12, are candidate biomarkers of systemic and myeloid-cell-associated inflammation in systemic JIA, but their diagnostic and prognostic utility depends on clinical context and requires interpretation alongside routine laboratory findings (53). MAS is a life-threatening hyperinflammatory complication of systemic JIA with a distinct pattern of clinical and laboratory abnormalities (54). In a multinational retrospective study of 362 patients with systemic JIA-associated MAS, fever, hepatomegaly, splenomegaly, central nervous system dysfunction, and hemorrhage were frequent clinical manifestations, while platelet count and levels of liver transaminases, ferritin, lactate dehydrogenase, triglycerides, and D-dimer were the laboratory measures that changed by more than 50% between the pre-MAS visit and MAS onset (54). A 2025 meta-analysis of 10 case-control studies including 1,936 children found that central nervous system involvement, hypofibrinogenemia, markedly elevated ferritin, and leukopenia were associated with MAS in systemic JIA (55). These findings provide Supportive evidence for risk stratification but do not establish causality; evidence for the standalone diagnostic or prognostic use of ferritin and S100 proteins remains Limited. Accordingly, adult RA biomarker frameworks centered on RF and ACPA have low transferability to systemic JIA, in which ferritin and S100 proteins should be interpreted as phenotype-specific inflammatory markers and MAS requires pediatric clinical and laboratory assessment.

In adult RA, persistent synovitis and structural damage are sustained by interactions among autoreactive lymphocytes, macrophages, fibroblast-like synoviocytes, and osteoclast-lineage cells (24, 56). Activated synovial fibroblasts amplify inflammatory signaling and promote RANKL-dependent osteoclastogenesis, thereby contributing to cartilage loss and bone erosion (24, 56). This immune–stromal–bone circuit is Established in adult RA, whereas direct pediatric evidence that the same tissue-destructive program dominates persistent arthritis in systemic JIA remains Limited. Adult RA mechanisms are therefore conditionally transferable to persistent arthritis in systemic JIA but have low transferability to the early systemic or MAS-associated phase.

### 6.2 RF-positive polyarticular JIA and adult seropositive RA: adaptive autoimmunity and erosive synovitis

RF-positive polyarticular JIA is the JIA phenotype most closely aligned with adult seropositive RA, but this relationship represents partial endotypic convergence rather than complete biological equivalence. In a genetic study of 340 patients with RF-positive polyarticular JIA and 14,412 controls, an adult RA weighted genetic risk score predicted RF-positive polyarticular JIA better than a score derived from oligoarticular or RF-negative polyarticular JIA loci, providing Supportive evidence of shared genetic susceptibility (18). Serologically, RF and ACPA identify an autoantibody-positive phenotype with greater potential for structural damage. In a retrospective study of 56 adults with persistent polyarticular-course JIA, RF positivity was associated with higher erosion and total Sharp–van der Heijde scores, while ACPA positivity was associated with greater erosion and total scores after adjustment for disease duration (4). Because this study involved a small adult cohort with longstanding disease, it provides Limited evidence for prognosis in children at diagnosis. B cells may contribute through autoantibody production, antigen presentation, cytokine release, and T-cell activation, but JIA-specific evidence remains less developed and is not consistently stratified by subtype (57).

The synovial compartment connects adaptive autoimmunity with local tissue damage. In adult RA, autoreactive lymphocytes, macrophages, fibroblast-like synoviocytes, and osteoclast-lineage cells form a tissue network in which inflammatory cytokines, RANKL, and matrix-degrading enzymes sustain synovitis and drive cartilage and bone damage (24). Within this Established RA framework, a recent translational study using RA patient samples, *in vitro* coculture experiments, and a collagen-induced arthritis mouse model found that IL-34 enhanced fibroblast-like synoviocyte–macrophage crosstalk, RANKL production, osteoclast differentiation, and bone erosion (58). Because this is a single mechanistic study without pediatric validation, IL-34 should be considered an Exploratory pathway rather than an established biomarker or therapeutic target. The broader immune–stromal–bone circuit is Established in adult RA, but evidence that the same cellular architecture and IL-34-dependent mechanism operate in RF-positive polyarticular JIA remains Limited.

RF-positive polyarticular JIA develops during skeletal growth, pubertal maturation, and age-dependent immune development. JIA-associated inflammation and its treatment can affect growth-plate function, bone modeling, and pubertal development, whereas immune maturation modifies how inflammatory arthritis emerges and is expressed across ages (9, 14). These developmental factors do not negate the genetic and serological overlap with adult seropositive RA, but they limit direct extrapolation of structural prognosis and tissue mechanisms from adults. Accordingly, adult seropositive RA evidence has High transferability for the broad genetic and autoantibody-associated disease model, Conditional transferability for the mechanisms and prognosis of synovitis and bone erosion, and Low transferability for outcomes that depend directly on growth-plate or pubertal biology.

### 6.3 Oligoarticular JIA: local pediatric synovial immunity

Oligoarticular JIA is poorly captured by adult RA-like RF/ACPA models and is better characterized by local synovial T-cell activation. Multi-omic profiling of synovial fluid from patients with oligoarticular JIA identified Th1 polarization across CD4+, CD8+, and γδ T-cell populations, with prominent Th1 skewing among CD4+ T cells and regulatory T cells and an association with severe disease (59). A separate single-cell study of JIA identified clonally expanded, PD-1-expressing tissue-resident memory-like T cells with pro-inflammatory features in synovial fluid (60). Because the latter cohort was not restricted to oligoarticular JIA, it supports a broader JIA synovial mechanism rather than a subtype-specific conclusion. Together, these studies provide Supportive evidence for a T-cell-centered model of oligoarticular synovitis, while the specific cellular states and their therapeutic implications remain Exploratory.

High-resolution studies have further characterized the cellular organization of pediatric synovitis. Single-cell RNA and T-cell receptor sequencing of three treatment-naïve patients with new-onset oligoarticular JIA, followed by validation in an independent mass-cytometry cohort, identified synovial enrichment and clonal expansion of CCL5-high GZMA-high effector-memory CD8+ T cells (61). A separate synovial tissue atlas of treatment-naïve JIA identified spatially organized immune and stromal niches associated with disease severity (10), while spatial transcriptomic analysis revealed endothelial–fibroblast and macrophage–CD8+ T-cell interactions and identified fibroblast states enriched in JIA compared with adult RA (11). Because the tissue-atlas and spatial-transcriptomic studies examined JIA more broadly and remain discovery-stage investigations, their findings cannot be assumed to be specific to oligoarticular JIA or interpreted as clinically validated biomarkers. These tissue-resolved findings are therefore Exploratory and support biological heterogeneity rather than a single established molecular target.

ANA positivity and susceptibility to uveitis form a clinically important component of the oligoarticular JIA phenotype. A systematic review and meta-analysis of 28 studies, including 5,902 patients with JIA, found substantial heterogeneity across JIA subtypes and ANA titres; at an ANA screening dilution of at least 1:40, pooled sensitivity and specificity for JIA-associated uveitis were 75% and 66%, respectively (62). A separate meta-analysis of 28 original studies involving 22,834 patients with JIA, including 3,381 who developed uveitis, identified younger age at JIA onset, ANA positivity, and elevated ESR as risk factors for uveitis (7). Current reviews describe the pathogenesis of JIA and JIA-associated uveitis as complex and incompletely understood (63). ANA should therefore be interpreted as a clinical risk-stratification marker rather than evidence of a single ANA-driven pathogenic pathway. Evidence for clinical risk stratification is Supportive, whereas evidence connecting ANA directly to the underlying joint and ocular mechanisms remains Limited. The RF/ACPA-centered adult RA model consequently has Low transferability to oligoarticular JIA.

### 6.4 Enthesitis-related arthritis and psoriatic JIA: mucosal–entheseal and skin-joint immune axes

Enthesitis-related arthritis (ERA) shifts the dominant inflammatory compartment away from classical RA-like synovitis. ERA belongs to the juvenile spondyloarthritis spectrum and is characterized by HLA-B27-associated susceptibility, peripheral arthritis and enthesitis, and possible sacroiliac or axial involvement (8). In adult spondyloarthritis, enthesis-centered inflammation and the IL-23/IL-17 axis are well described, but the available evidence does not establish that the same pathway is uniformly dominant in pediatric ERA (64). Pediatric ERA-specific mechanistic evidence remains Limited. Adult spondyloarthritis evidence therefore has Conditional transferability to ERA, whereas the RF/ACPA-centered adult RA model has Low transferability.

Evidence for a gut-joint contribution to ERA remains Exploratory. In a cross-sectional study of 15 patients with JIA-ERA, including 10 HLA-B27-positive and 5 HLA-B27-negative patients, and 10 healthy controls, HLA-B27-positive patients had higher fecal calprotectin levels than both HLA-B27-negative patients and healthy controls (49). Serum lipopolysaccharide levels did not differ, and alpha and beta diversity were similar between the HLA-B27-positive and HLA-B27-negative groups, although several bacterial taxa differed between groups (49). The small sample, cross-sectional design, and multiple taxonomic comparisons preclude causal inference and limit generalization. These findings support further investigation of a mucosal-entheseal model but do not validate microbiome composition or fecal calprotectin as causal, diagnostic, or treatment-selection biomarkers in ERA.

Juvenile psoriatic arthritis is heterogeneous and should not be treated as a single RA-like synovial phenotype. Reviews distinguish an early-onset phenotype, often ANA-positive and overlapping clinically with other young-onset JIA categories, from an older-onset phenotype with enthesitis, possible axial involvement, and greater resemblance to adult psoriatic arthritis (23). Proposed skin–joint and entheseal mechanisms involving dendritic cells and IL-17/IL-23 signaling are derived largely from psoriasis and adult psoriatic arthritis, whereas pediatric genetic and mechanistic data remain sparse (23). A recent treatment review similarly found that many therapies targeting IL-17, IL-12/23, IL-23, JAK, and related pathways have been evaluated primarily in adults, leaving important translational gaps in juvenile psoriatic arthritis (65). The pediatric mechanistic evidence is therefore Limited, adult psoriatic arthritis evidence has Conditional transferability, and the adult RA RF/ACPA model has Low transferability.

### 6.5 Adult RA as a comparator: adaptive autoimmune synovitis and joint destruction

Adult seropositive RA provides the closest adult comparator for RF-positive polyarticular JIA. In RA, loss of immune tolerance and autoantibody-associated adaptive immune responses converge on chronic synovitis, while interactions among immune cells, synovial fibroblasts, and osteoclast-lineage cells promote cartilage and bone damage (66, 67). Genetic evidence indicates that RF-positive polyarticular JIA is more closely related to adult RA than oligoarticular or RF-negative polyarticular JIA (18), and RF and ACPA positivity are associated with greater structural damage in polyarticular-course JIA persisting into adulthood (4). This correspondence is strongest for genetic and autoantibody-associated features but remains incomplete at the level of pediatric synovial tissue mechanisms. Adult RA mechanisms may be partly applicable to persistent afebrile arthritis in systemic JIA but poorly capture systemic and MAS-associated disease, oligoarticular JIA, enthesitis-related arthritis, or juvenile psoriatic arthritis, in which different inflammatory compartments and immune pathways predominate. Adult RA therefore serves as a phenotype- and domain-specific comparator rather than a universal biological model of childhood arthritis (**Figure 2**).

**Figure 2:**
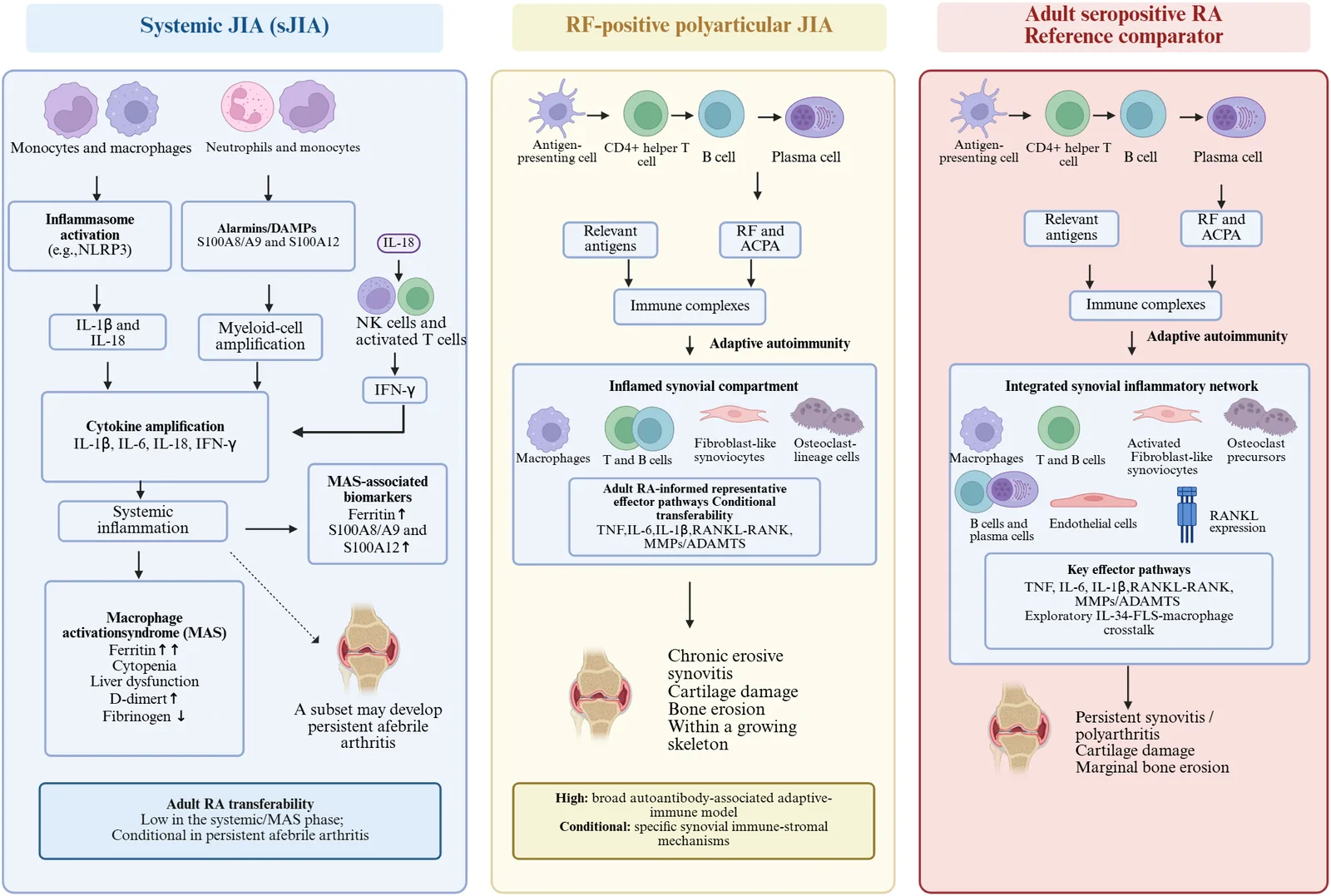
Comparative immune mechanisms in systemic juvenile idiopathic arthritis, rheumatoid factor-positive polyarticular juvenile idiopathic arthritis, and adult seropositive rheumatoid arthritis. Systemic JIA is characterized predominantly by myeloid-cell activation and cytokine amplification, with macrophage activation syndrome representing a major hyperinflammatory complication. Adult RA mechanisms have Low transferability to the systemic/MAS phase but Conditional transferability to persistent afebrile arthritis. RF-positive polyarticular JIA shows High transferability for the broad autoantibody-associated adaptive-immune model and Conditional transferability for specific synovial immune–stromal mechanisms.

## 7 Biomarkers: clinical function, evidence maturity, and transferability

For clarity, diagnostic biomarkers support disease recognition or classification; prognostic biomarkers estimate the future disease course irrespective of treatment; predictive biomarkers estimate the likelihood of response to a specific intervention; disease-activity biomarkers reflect the current inflammatory burden; and pharmacodynamic biomarkers change after treatment exposure and indicate a biological response without necessarily predicting pretreatment benefit.Biomarkers in JIA differ in clinical function and cannot be interpreted as a single category. RF, ACPA, ANA, and HLA-B27 are informative only within specific phenotypes, whereas ferritin, S100 proteins, cytokines, and tissue- or omics-derived signatures reflect different aspects of systemic inflammation, local tissue activity, prognosis, or treatment response. A biological association alone does not establish clinical utility; application requires reproducible assays, standardized cut-offs, appropriate estimates of diagnostic or predictive performance, and independent validation in age- and phenotype-defined cohorts. These requirements are particularly important when biomarkers developed in adult RA are considered for JIA.

### 7.1 Conventional and subtype-linked biomarkers

RF and ACPA should be interpreted according to their clinical function rather than as general biomarkers of JIA. In adult RA, both are established classification and prognostic biomarkers. Across studies summarized in a major review, RF showed a reported sensitivity of 62-87% and specificity of 43-96% for RA, whereas ACPA showed a sensitivity of 41-77% and specificity of 88-98%; combined RF and ACPA positivity had a reported sensitivity of 33-57% and specificity of 91-99% (68). Under the ILAR classification criteria, RF positivity documented on at least two occasions at least three months apart during the first six months is category-defining for RF-positive polyarticular JIA, whereas ACPA is not included as a category-defining marker and has been investigated mainly for structural-risk stratification (4, 69). In a retrospective study of 56 adults with polyarticular JIA persisting into adulthood, RF positivity was associated with higher erosion and total Sharp–van der Heijde scores. After adjustment for disease duration, ACPA positivity was associated with higher erosion and total scores, and ACPA levels correlated with the total structural-damage score (r=0.54) (4). This study provides Limited evidence for structural prognosis because it involved a small retrospective adult cohort with persistent disease; diagnostic performance at childhood onset, pediatric cut-offs, and independent pediatric validation were not evaluated. Adult RA evidence therefore has High transferability for recognizing a broad RF-positive, autoantibody-associated phenotype and Conditional transferability for estimating structural risk during childhood. Transferability for treatment selection is Low because current evidence does not validate either RF or ACPA for selecting a specific therapy in JIA. These similarities do not establish complete biological equivalence.

In systemic JIA, ferritin, IL-18, S100A8/A9 (calprotectin), and S100A12 should be interpreted primarily as disease-activity and MAS-associated hyperinflammation biomarkers rather than as RA-like diagnostic autoantibodies or validated treatment-predictive markers (53). In a cross-sectional cohort of 40 patients, total serum IL-18 distinguished active from clinically inactive disease with an area under the receiver-operating-characteristic curve of 0.8145 and study-derived sensitivity and specificity of 76.2% and 84.2%, respectively; corresponding values for distinguishing patients with versus without a history of MAS were 0.84, 85%, and 70% (70). However, only two patients had active MAS at sampling, IL-18 remained elevated in 79% of patients with clinically inactive disease, and the reported cut-offs have not been independently validated. The evidence is therefore Supportive for an association with systemic and myeloid inflammatory activity but Limited for prospective prediction of incident MAS, stand-alone diagnosis, or treatment selection. Because these biomarkers reflect Still disease and MAS-related inflammation rather than autoantibody-associated adult RA synovitis, transferability from the adult RA biomarker framework is Low.

ANA should be interpreted primarily as an ocular-risk stratification biomarker in JIA rather than as a diagnostic marker for JIA or a marker of current articular disease activity. In a systematic review and meta-analysis of 28 studies including 5,902 patients with JIA, ANA at a screening dilution of ≥1:40 had a pooled sensitivity of 75% (95% CI 46-100%) and specificity of 66% (95% CI 39-93%) for JIA-associated uveitis, with substantial heterogeneity across JIA subtypes and ANA titres (62). A separate meta-analysis of 28 studies including 22,834 patients found that ANA positivity was associated with uveitis (OR 1.993, 95% CI 1.726-2.301); each additional year of age at JIA onset was associated with a lower risk (OR 0.860, 95% CI 0.809-0.914), whereas higher ESR was associated with a higher risk (OR 1.025 per 1 mm/h, 95% CI 1.003-1.048) (7). Evidence is therefore Established for the association of ANA positivity with increased ocular risk at the population level, but Limited for individualized prediction because screening dilutions varied, between-study heterogeneity was substantial, and pooled positive and negative predictive values were not reported. ANA is not established as a treatment-predictive or pharmacodynamic biomarker. Because its principal clinical role is specific to pediatric uveitis risk, transferability from the adult RA biomarker framework is Low.

In enthesitis-related arthritis (ERA), HLA-B27 functions primarily as a classification-supporting and candidate prognostic risk-stratification marker rather than as a stand-alone diagnostic, disease-activity, or treatment-response biomarker. Under the ILAR criteria, ERA is defined by arthritis and enthesitis, or by arthritis or enthesitis plus at least two additional features, one of which may be HLA-B27 positivity; HLA-B27 alone is therefore insufficient for classification (69). In the updated population-based Nordic JIA cohort, HLA-B27 status was available for 416 of 434 participants and was positive in 93 (22.4%). After 18 years, HLA-B27 positivity was associated with a higher risk of not being in remission off medication (OR 2.6, 95% CI 1.5–4.3), with a stronger association in men (OR 5.6) (71). These findings support long-term risk stratification but do not establish individual predictive performance, because spondyloarthritis-related clinical manifestations were themselves associated with poorer remission outcomes. The study did not report sensitivity, specificity, positive predictive value, or negative predictive value for ERA diagnosis or individual outcome prediction. Evidence is therefore Established for the classification-supporting role of HLA-B27 and Supportive for long-term prognostic risk stratification, but insufficient for stand-alone diagnosis or treatment selection. Because HLA-B27 belongs to the spondyloarthritis rather than autoantibody-associated RA framework, transferability from adult RA is Low, and adult spondyloarthritis is the more relevant adult comparator (8).

### 7.2 Source-specific and emerging biomarkers

Emerging biomarker research in JIA extends beyond conventional laboratory markers to calprotectin, microRNAs, proteomic and metabolomic profiles, and tissue-resolved cellular signatures (11). These candidates have been investigated for disease characterization, activity monitoring, prognosis, and treatment-response stratification, but most lack standardized cut-offs, longitudinal validation, and independent confirmation across JIA phenotypes (12). Adult RA provides a more mature model of molecular biomarker development, including synovial signatures evaluated against treatment response, although adult-derived signatures cannot be assumed to apply directly to pediatric arthritis (72).

Synovial fluid provides tissue-proximal molecular information, but the reported cellular signals should be interpreted as exploratory endotypic features rather than clinically validated biomarkers. Vanni et al. enrolled 22 children with JIA for peripheral-blood and synovial-fluid sampling and re-analysed existing single-cell RNA and T-cell receptor data derived from the blood and synovial fluid of seven patients; the principal single-cell comparison involved oligoarticular JIA samples and identified clonally expanded PD-1-expressing CD4 and CD8 T-cell states with tissue-resident memory-like and pro-inflammatory features in synovial fluid (60). Because the study did not relate these cellular states to a prespecified diagnostic, prognostic, disease-activity, or treatment-response endpoint, it provides mechanistic rather than clinical biomarker evidence. In a separate discovery study, single-cell RNA and T-cell receptor sequencing of paired blood and synovial fluid from three treatment-naïve patients with new-onset oligoarticular JIA identified synovial enrichment and clonal expansion of CCL5-high/GZMA-high effector-memory CD8 T cells (61). An independent mass-cytometry cohort included 13 patients with oligoarticular JIA, eight patients with uveitis flare, and six healthy controls; however, the reported validation principally confirmed alterations in circulating immune-cell populations rather than independently reproducing the synovial-fluid signature (61). Neither study reported standardized cut-offs, sensitivity, specificity, predictive values, longitudinal outcome discrimination, or treatment-specific predictive performance. The evidence is therefore Exploratory for tissue-based endotypic characterization. The methodological concept of tissue-proximal profiling is conditionally transferable from adult RA, but the specific cellular signatures require pediatric, phenotype-specific longitudinal validation.

Synovial tissue profiling can identify local cellular and molecular states that are not reliably represented by peripheral blood or synovial fluid, but current pediatric studies remain at the discovery stage (10, 11). In a multimodal study of 19 DMARD-naïve children with early JIA, single-cell RNA sequencing was performed on synovial tissue from 10 participants and spatial transcriptomics on tissue from eight; the analysis identified spatially organized immune–stromal niches and mapped genes previously associated with a more extensive disease course predominantly to SPP1-positive MERTK-negative CD206-negative macrophages (10). However, this severity-associated gene set was derived from an earlier synovial-fluid study comparing eight children whose oligoarticular JIA subsequently extended with 13 whose disease remained persistent, rather than being prospectively validated against outcomes in the biopsy cohort (10, 73). A separate spatial transcriptomic study analyzed active synovitis from nine patients with RF-negative oligoarticular or polyarticular JIA, of whom three were treatment-naïve and six had received heterogeneous therapies (11). The study identified predicted endothelial–fibroblast and macrophage–CD8-positive T-cell interactions, tertiary lymphoid structures, and JIA-enriched NOTCH3-positive and CXCL12-positive sublining fibroblast states relative to adult RA (11). However, no statistically significant associations between synovial cell populations and the tested clinical covariates other than CRP levels were identified, and the small, treatment-heterogeneous cohort and indirect cross-study comparison with adult RA limit generalizability (11). Neither study reported predefined cut-offs, diagnostic performance, longitudinal outcome discrimination, treatment-response prediction, or independent clinical validation (10, 11). The mechanistic evidence supports spatially organized JIA synovitis, but tissue-derived signatures remain Exploratory as clinical biomarkers. Adult RA tissue-profiling methods can inform pediatric study design, although the specific JIA signatures cannot yet support diagnosis, prognosis, or treatment selection.

Outside systemic JIA, serum calprotectin (S100A8/A9) has been investigated primarily as a monitoring and prognostic marker rather than as a stand-alone diagnostic test. In a prospective longitudinal cohort of 60 patients with non-systemic JIA in clinical remission, 31 (51.6%) experienced relapse within 18 months. Higher baseline serum calprotectin was associated with relapse at 18 months (OR 1.66, 95% CI 1.13-2.44), whereas a higher baseline musculoskeletal ultrasound score was associated with relapse at six months (OR 1.96, 95% CI 1.09-3.52) (74). This study provides Supportive evidence for an association with unstable remission, but the mixed non-systemic JIA population, single-center design, and lack of an externally validated threshold or prediction model limit the use of serum calprotectin as a stand-alone relapse predictor. In an exploratory analysis of a phase 3 abatacept trial in polyarticular-course JIA, baseline biomarker samples were available for 158 of 219 participants. Lower baseline S100A8/A9 levels (≤3295 ng/mL) and S100A12 levels (≤176 ng/mL) were associated with greater odds of achieving JIA-ACR inactive disease at month 4, with ORs of 4.25 (95% CI 2.03-8.92) and 3.66 (95% CI 1.76-7.61), respectively (75). However, this was a treatment-specific exploratory analysis, the thresholds were derived from the study dataset, and the findings were not independently validated. The evidence is therefore Limited for treatment-response prediction. Adult RA findings on calprotectin have Conditional biological and methodological transferability, but pediatric phenotype-specific thresholds and prospective validation are required before routine relapse prediction or treatment selection.

Beyond conventional ANA positivity, emerging serological studies in JIA-associated uveitis have examined ANA titers, HEp-2 indirect immunofluorescence patterns, and anti-prefoldin subunit 5 antibodies as potential tools for ocular-risk stratification. ANA positivity has an established role in population-level ocular-risk stratification, although its performance is insufficient for stand-alone individual prediction (7, 62). In a convenience sample from the prospectively followed ICON inception cohort that was enriched for uveitis cases, 52 patients with uveitis and 141 without uveitis were analyzed (76). ANA titers were higher in patients with uveitis than in those without uveitis (p<0.001), and a study-derived threshold of ≥1:320 yielded 75% sensitivity and 62% specificity (76). The AC-30 indirect immunofluorescence pattern was more frequent in patients with uveitis than in those without uveitis (63.5% vs. 23.4%; p<0.001), while anti-prefoldin subunit 5 antibodies were detected in 44% and 21%, respectively (p=0.009) (76). A model based on age at JIA onset, ILAR category, and sex had an area under the receiver-operating-characteristic curve of 0.77; adding ANA titer, anti-prefoldin subunit 5 antibodies, and the AC-30 and AC-4 patterns increased the area to 0.86, with an integrated discrimination improvement of 0.06 (p=0.005) (76). However, the reported signals primarily concerned uveitis occurrence; higher ANA titers and the AC-30 pattern were not associated with uveitis-related complications. Convenience sampling, inclusion of only uveitis occurring within three years of JIA diagnosis, possible effects of early DMARD exposure, absence of longitudinal antibody measurements, and lack of independent external validation limit clinical application. Evidence is therefore Established for the association of ANA positivity with increased ocular risk, but Limited for individualized prediction based on higher ANA titers or the AC-30 pattern and Exploratory for anti-prefoldin subunit 5 antibodies. Because this risk profile concerns a pediatric extra-articular complication without a direct adult RA counterpart, transferability from the adult RA biomarker framework is Low. These emerging biomarkers should not replace scheduled slit-lamp screening.

In ERA, plasma MRP8/14 (S100A8/A9) and serum MMP-3 have been investigated as disease-activity and monitoring biomarkers, whereas fecal calprotectin should be interpreted as a surrogate marker of intestinal rather than articular inflammation. In a single-center study of 69 patients with ERA, plasma MRP8/14 levels were higher in active than inactive disease (median 11,669.5 vs. 4,421.8 ng/mL; p<0.0001) and decreased after three months only in treatment responders (p=0.012) (77). In a separate cohort of 54 patients, serum MMP-3 correlated with tender and swollen joint counts, ESR, BASDAI, BASFI, pain, and patient and physician global assessments; among 24 longitudinally followed patients, changes in MMP-3 correlated with changes in tender and swollen joint counts (78). In a cross-sectional exploratory study of 71 participants, fecal calprotectin was highest in ERA and was higher in active than inactive ERA (median 57.4 vs. 20.0 mg/kg; p=0.01) and in patients with versus without MRI-detected sacroiliitis (54.3 vs. 22.6 mg/kg; p=0.04) (79). A later small study of 10 HLA-B27-positive and five HLA-B27-negative patients with JIA-ERA and 10 healthy controls found higher fecal calprotectin in the HLA-B27-positive group, whereas serum lipopolysaccharide did not differ and alpha and beta diversity were similar between the two ERA groups (49). None of these studies established standardized clinical cut-offs, sensitivity, specificity, predictive values, or independent prospective validation. Evidence is therefore Supportive for associations of MRP8/14 and MMP-3 with disease activity, Limited for serial treatment monitoring, and Exploratory for fecal calprotectin and HLA-B27-stratified intestinal inflammation. Because this biomarker profile belongs to the spondyloarthritis rather than autoantibody-associated RA spectrum, transferability from the adult RA biomarker framework is Low; adult spondyloarthritis is the more relevant adult comparator.

In adult RA, emerging biomarker studies have advanced from biological associations toward treatment-specific molecular prediction, although few tools have established prospective clinical utility. In STRAP, pretreatment synovial RNA models predicted 16-week response to etanercept, tocilizumab, and rituximab, with cross-validated areas under the curve of approximately 0.75; the tocilizumab and rituximab models were externally evaluated in R4RA, and a 524-gene synovial panel achieved areas of 0.82–0.87 in residual STRAP samples (72). R4RA further linked humoral immune programs to drug response and a stromal–fibroblast program to multidrug-refractory disease, although predictive performance remained moderate (80). Blood-based neutrophil-activation and metabolomic signatures have also been associated with baricitinib response or remission, but treatment specificity and modest discrimination limit their generalizability (81, 82). The molecular signature response classifier showed a positive predictive value of 87.7%, sensitivity of 60.2%, and specificity of 77.3% for TNF-inhibitor non-response (83), while subsequent classifier-informed care was associated with better outcomes than matched external controls but was not evaluated in a randomized comparison (84). Evidence is therefore Supportive for molecular stratification in adult RA but Limited for routine treatment selection. Methodological transferability to JIA is Conditional, whereas direct transferability of adult-derived signatures is Low.

Taken together, emerging biomarker evidence is more mature in adult RA than in JIA, although clinical readiness remains uneven in both diseases. In JIA, tissue and molecular studies mainly define phenotype-specific inflammatory endotypes, whereas adult RA studies have progressed further toward treatment-specific prediction. Most emerging JIA candidates therefore remain Limited or Exploratory for clinical use, and age-stratified performance, inter-assay reproducibility, and longitudinal stability have rarely been evaluated. Adult RA biomarker-development methods have Conditional transferability to JIA, whereas direct transferability of adult-derived signatures is Low. Pediatric candidates require validation for a predefined clinical function in age- and phenotype-specific cohorts before they can complement established subtype-linked biomarkers, clinical assessment, and imaging.

### 7.3 Treatment-response biomarkers and current limitations

Clinical translation of emerging biomarkers in JIA is limited primarily by small discovery cohorts, phenotype heterogeneity, and a lack of independent longitudinal validation (10, 61, 75, 85). Tissue-proximal profiling can reveal local inflammatory states that are not fully represented in peripheral blood, but repeated sampling of synovial fluid or tissue is impractical in children (10, 60, 61). Clinical validation requires prespecified age and phenotype strata, evaluation of disease activity and treatment effects on biomarker concentrations, assessment of inter-assay and inter-platform reproducibility, and clinically relevant cut-offs. Some adult RA molecular signatures have undergone independent external validation or prospective interventional evaluation (72, 84), whereas JIA biomarker studies remain largely exploratory or *post hoc* and have not shown that biomarker-guided treatment selection improves clinical outcomes (75, 85). These limitations prevent unqualified extrapolation across JIA phenotypes and limit direct transfer of adult RA-derived signatures. Evidence for routine biomarker-guided treatment selection in JIA therefore remains Limited.

Candidate treatment-response biomarkers in JIA fall into two groups: pretreatment predictive markers and on-treatment pharmacodynamic markers. In polyarticular-course JIA, lower pretreatment S100A8/A9 and S100A12 levels were associated with deeper clinical responses among abatacept-treated patients. However, because this was a post-hoc analysis of a single-arm, open-label study, it could not establish treatment-specific predictive utility, and the study-derived thresholds were not independently validated (75). In a single-center prospective observational cohort of 60 patients initiating methotrexate, pretreatment plasma hippurate was independently associated with non-response at six months and showed an area under the receiver-operating-characteristic curve of 0.781; the multi-platform metabolomic discovery design and lack of external validation make this finding Exploratory (86). By contrast, the JUVE-BASIS post-hoc analysis measured serum-protein changes during the 12-week open-label baricitinib lead-in period; correlations between changes in CCL7, CCL18, IL-6, and MMP-3 and clinical response therefore represent pharmacodynamic associations rather than validated pretreatment prediction (85). Prospective clinical utility for assigning one JIA therapy over another has not been demonstrated. Evidence is therefore Limited for an association between baseline S100 levels and response under abatacept and Exploratory for methotrexate-response prediction and baricitinib pharmacodynamic monitoring; treatment selection remains driven primarily by clinical phenotype, disease activity, previous treatment exposure, and safety (**Table 2**).

**Table 2:** Conventional and emerging biomarkers, clinical interpretation, and limitations across representative juvenile idiopathic arthritis subtypes and adult rheumatoid arthritis.

Representative JIA subtype/adult RA	Conventional biomarkers	Emerging biomarkers	Clinical interpretation and potential utility	Main limitations	References
Systemic JIA	Ferritin, ESR, CRP	IL-18, S100A8/A9 (calprotectin), S100A12	IL-18 concentrations are higher in active systemic JIA and in patients with a history of MAS, supporting an association with MAS-related hyperinflammation; S100A8/A9 and S100A12 reflect neutrophil/myeloid activation and disease activity.	Phenotype-dependent interpretation is required, and assays, thresholds, and sampling schedules are not standardized. Prospective MAS prediction, incremental value beyond ferritin and routine inflammatory markers, and independent external validation remain limited.	(53, 70)
RF-positive polyarticular JIA	RF, ACPA, ESR, CRP	S100A8/A9 and S100A12 as candidate treatment-response biomarkers for abatacept in polyarticular-course JIA; plasma metabolomic signatures associated with methotrexate non-response in broader JIA cohorts	Lower baseline S100A8/A9 and S100A12 concentrations were associated with better abatacept response in polyarticular-course JIA. Metabolomic profiles have also been associated with methotrexate non-response in broader JIA cohorts, but neither finding has validated predictive utility specifically in RF-positive polyarticular JIA.	Treatment-response evidence is exploratory, derives from polyarticular-course or broader JIA cohorts, and lacks predefined cutoffs and independent prospective validation specifically in RF-positive polyarticular JIA.	(4, 69, 75, 86)
Oligoarticular JIA	ANA, ESR, CRP	Higher ANA titres and the AC-30 pattern; anti-prefoldin 5 antibodies; synovial-fluid PD-1^+^ T cells and CCL5^Hi^ GZMA^Hi^ effector-memory CD8 T cells	High ANA titres and AC-30 pattern may refine ocular risk; anti-prefoldin 5 is a candidate biomarker. Synovial PD-1+ T-cell enrichment and CCL5Hi GZMAHi CD8 signatures indicate local immune activation but are not established clinical tests.	Candidate serological markers require independent validation; systemic markers may not fully capture localized synovitis or uveitis, and synovial-fluid sampling is difficult in children.	(7, 60, 61, 76)
Enthesitis-related arthritis	HLA-B27, ESR, CRP	Plasma MRP8/14 (S100A8/A9); serum matrix metalloproteinase-3 (MMP-3); fecal calprotectin	Plasma MRP8/14, serum MMP-3, and fecal calprotectin are elevated in active ERA/sacroiliitis, with MRP8/14 decreasing in treatment responders.	Evidence is based mainly on small, single-center cohorts; MRP8/14 and MMP-3 are not specific to ERA, and fecal calprotectin reflects intestinal rather than articular inflammation. Subtype-specific cutoffs and independent prospective validation are lacking.	(8, 49, 77, 78, 112)
Juvenile psoriatic arthritis	ANA, ESR, CRP; HLA-B27 in patients with enthesitis or axial features	No subtype-specific emerging biomarker has been independently validated for routine clinical use	ANA may aid uveitis-risk assessment in younger patients, whereas HLA-B27 can support characterization of enthesitis or axial involvement; neither is diagnostic of juvenile psoriatic arthritis. Biomarkers currently assist phenotype and complication assessment rather than treatment-response selection.	Juvenile psoriatic arthritis is heterogeneous, psoriasis may appear after arthritis onset, and pediatric studies often aggregate this subtype with other JIA or juvenile spondyloarthritis categories. Subtype-specific cutoffs and prospective treatment-response validation are lacking.	(23, 69)
Adult rheumatoid arthritis	RF, ACPA, ESR, CRP	Synovial B-cell/humoral and stromal-fibroblast markers (CD20, CD138, CD68, CD3, and DKK3/CD90); plasma calprotectin and neutrophil extracellular traps (NETs), with whole-blood FCAR (FcαRI/CD89) transcripts	Baseline synovial RNA and B-cell/stromal signatures demonstrated treatment-specific predictive performance for etanercept, tocilizumab, or rituximab in selected cohorts. Neutrophil-activation markers were associated with baricitinib response, exploratory metabolomic profiles were associated with treatment response in early RA, and the MSRC was developed to identify inadequate TNFi response.	Synovial biopsy is invasive, and synovial and blood signatures remain treatment-, platform-, and cohort-specific. Metabolomic associations were exploratory and showed only modest predictive discrimination. The MSRC addresses inadequate TNFi response but does not select a specific alternative therapy; routine clinical utility requires further independent prospective and comparative validation.	(68, 72, 80–84)

ACPA, anti-citrullinated protein antibodies; AC-30, fine speckled with mitotic plate antinuclear antibody pattern; ANA, antinuclear antibodies; CCL5, C-C motif chemokine ligand 5; CD, cluster of differentiation; CRP, C-reactive protein; DKK3, Dickkopf-related protein 3; ERA, enthesitis-related arthritis; ESR, erythrocyte sedimentation rate; FCAR, Fc alpha receptor gene; FcαRI, Fc alpha receptor I; GZMA, granzyme A; HLA-B27, human leukocyte antigen B27; IL-18, interleukin-18; JIA, juvenile idiopathic arthritis; MAS, macrophage activation syndrome; MMP-3, matrix metalloproteinase-3; MRP8/14, myeloid-related proteins 8 and 14; MSRC, molecular signature response classifier; NETs, neutrophil extracellular traps; PD-1, programmed cell death protein 1; RA, rheumatoid arthritis; RF, rheumatoid factor; S100A8/A9, S100 calcium-binding proteins A8 and A9 (calprotectin); S100A12, S100 calcium-binding protein A12; TNFi, tumor necrosis factor inhibitor. Superscript “Hi” denotes high expression, and “+” denotes marker-positive cells.

Only representative JIA subtypes and adult RA are shown; this table is not intended to provide a complete classification of JIA.

## 8 Treatment strategies by age, subtype, and target

### 8.1 Shared therapeutic principles and age-specific considerations

JIA and adult RA share the therapeutic goals of suppressing inflammation, preventing irreversible structural damage, preserving function, and improving quality of life. In adult RA, the 2025 EULAR recommendations retain methotrexate as the initial anchor DMARD, ideally combined with short-term glucocorticoids that should be tapered and discontinued as rapidly as clinically feasible. If the treatment target is not achieved within 3–6 months, a bDMARD should be added, whereas a JAK inhibitor may be considered after individualized assessment of the risks of major adverse cardiovascular events, malignancy, and thromboembolic events (87). Compared with the 2022 update, the 2025 recommendations remove stratification according to poor prognostic factors after failure of the initial csDMARD strategy: an inadequate response now supports addition of a bDMARD, although another csDMARD may remain an option in selected settings. After failure of a first bDMARD or JAK inhibitor, another bDMARD from the same or a different class, or a JAK inhibitor after renewed risk assessment, may be used. After glucocorticoids have been discontinued and sustained remission has been achieved, continuation of DMARDs is recommended, although dose reduction may be considered; complete withdrawal frequently leads to disease flare (87). Treat-to-target principles have also been proposed and increasingly studied in pediatric rheumatic diseases (88). In JIA, the 2026 ACR guideline summary supports early phenotype-directed treatment: IL-1 or IL-6 inhibition is recommended as first-line therapy for newly diagnosed systemic JIA, consistent with the 2024 EULAR/PReS recommendations for Still’s disease (6, 89); intra-articular glucocorticoids are recommended as first-line therapy for oligoarthritis, whereas csDMARD and/or bDMARD therapy is recommended as first-line disease-modifying treatment for polyarthritis, enthesitis, dactylitis, and temporomandibular joint arthritis (89). Although several of these pediatric recommendations are strong, the underlying evidence is frequently of low or very low certainty; recommendation strength should therefore not be equated with evidence quality or interpreted as evidence of biological equivalence across JIA phenotypes (89). Because labeled indications and minimum treatment ages vary across jurisdictions, guideline recommendations should not be interpreted as uniform regulatory authorization. Adult RA treatment principles are therefore most informative for RF-positive polyarticular JIA, but their transferability remains Conditional across the broader JIA spectrum.

Shared therapeutic targets do not imply identical monitoring requirements or long-term risk-benefit balances across age groups. In children and adolescents, active inflammation and glucocorticoid exposure can impair linear growth, pubertal progression, and bone accrual (9). Prolonged glucocorticoid treatment may also suppress the hypothalamic-pituitary-adrenal axis (90). The 2026 ACR guideline summary recommends baseline and medication-specific laboratory monitoring, regular assessment of growth and bone health during chronic systemic glucocorticoid treatment, screening for mental health concerns, and formal assessment of transition readiness, although many of these recommendations remain supported by low- or very-low-certainty evidence (89). Vaccination status and timing should be reviewed before and during immunosuppressive therapy. Non-live vaccines should generally be administered according to national schedules, whereas live-attenuated vaccines are usually avoided during immunosuppression, with guideline-defined exceptions considered individually (91). Long-term pediatric safety data for biologic therapies, derived largely from registries of polyarticular-course JIA, remain incomplete for rare or delayed outcomes, and continued surveillance is required for serious infections, autoimmune complications, and malignancy during prolonged exposure (92). Pediatric experience with JAK inhibitors is more recent and likewise requires long-term vigilance for potential cumulative or delayed harms (93). Age-appropriate reproductive counseling, adherence support, psychosocial assessment, and coordinated transition beginning in early adolescence should be incorporated into care across pediatric and adult services (94). These developmental and safety considerations mean that use of the same molecular target or drug class in JIA and adult RA does not imply an identical monitoring strategy or risk-benefit balance.

### 8.2 Systemic JIA: cytokine-directed therapy and macrophage activation syndrome

Systemic JIA is increasingly managed within the broader Still’s disease framework, which also encompasses adult-onset Still’s disease, rather than through an adult RA-like csDMARD step-up model (6). Treatment targets include rapid control of systemic inflammation, prompt recognition and management of macrophage activation syndrome (MAS), achievement of clinically inactive disease, and minimization of glucocorticoid exposure. The 2024 EULAR/PReS recommendations prioritize IL-1 or IL-6 inhibition and advise initiation as early as possible after diagnosis, without requiring prior failure of NSAIDs or csDMARDs (6). The 2026 ACR guideline summary likewise strongly recommends first-line IL-1 or IL-6 inhibition for newly diagnosed systemic JIA both without and with MAS, although the guideline rates the supporting evidence as being of low certainty (89). The unified Still’s disease framework supports shared cytokine-directed treatment principles, while pediatric dosing, safety, developmental context, and complication surveillance remain age-specific. Adult RA drug-sequencing principles therefore have Low transferability during cytokine-dominant systemic disease and become Conditional only when systemic manifestations are controlled and persistent arthritis becomes the predominant treatment target. The 2026 ACR guideline summary conditionally recommends routine screening for systemic JIA-associated lung disease and strongly recommends that the presence or development of lung disease should not be considered an absolute contraindication to IL-1 or IL-6 inhibition (89).

Guidance on NSAID and glucocorticoid use in systemic JIA has shifted toward earlier cytokine-directed therapy. The 2021 ACR guideline conditionally allowed a short trial of NSAID monotherapy in newly diagnosed systemic JIA without MAS, based on very-low-certainty evidence, but advised rapid escalation if the response was not rapid and complete (95). By contrast, the 2026 ACR guideline summary strongly recommends against NSAID monotherapy, although the underlying evidence remains of very low certainty (89). The EULAR/PReS recommendations limit NSAIDs to symptomatic management of fever and arthralgia during diagnostic evaluation, and their use should not delay cytokine-directed treatment once systemic JIA is established (6). Outside MAS, systemic glucocorticoids should be used only as short-duration adjunctive therapy and tapered as intermediate treatment targets are achieved (6). When MAS is present, the 2026 ACR guideline summary strongly recommends first-line IL-1 or IL-6 inhibition and also strongly recommends systemic glucocorticoids as part of initial therapy (89). For active MAS that does not respond to or is not tolerated with the initial bDMARD, the guideline conditionally favors another bDMARD or tsDMARD over adding cyclosporine or tacrolimus, although the evidence is of very low certainty (89). By comparison, the EULAR/PReS recommendations identify high-dose glucocorticoids as the basis of MAS treatment and support consideration of anakinra, cyclosporine, and/or interferon-γ inhibition according to disease severity, with expert-center collaboration for severe, recurrent, or difficult-to-treat disease (6). This difference reflects variation in guideline scope and very-low-certainty evidence rather than direct comparative evidence establishing a single preferred rescue sequence.

Conventional csDMARDs should not be used as initial monotherapy for the active systemic manifestations of systemic JIA. In the systemic JIA subgroup of the only randomized placebo-controlled trial cited by the EULAR/PReS task force, the JIA-ACR30 response was not significantly different between methotrexate and placebo (25% vs. 16%) (6), and the 2026 ACR guideline summary retains a strong recommendation against initial csDMARD monotherapy, although the supporting evidence is of low certainty (89). Subsequent treatment should be guided by the dominant residual disease domain. For ongoing systemic manifestations despite an inadequate response or intolerance to an initial bDMARD, a different IL-1 or IL-6 inhibitor or a JAK inhibitor is conditionally preferred over adding methotrexate or systemic glucocorticoids, with IL-1 or IL-6 inhibition preferred over JAK inhibition (89). When systemic manifestations are controlled but residual arthritis persists during bDMARD therapy, changing targeted therapy, adding a csDMARD such as methotrexate, or using intra-articular glucocorticoids is conditionally recommended over adding systemic glucocorticoids, although the evidence is of very low certainty (89). Methotrexate therefore has a secondary, arthritis-directed role in systemic JIA rather than the anchor-DMARD role it occupies in adult RA (87).

### 8.3 RF-positive polyarticular JIA: therapeutic overlap with adult seropositive RA

Among the JIA phenotypes, RF-positive polyarticular JIA has the closest genetic relationship to adult seropositive RA (18). In a retrospective cohort of 56 adults with polyarticular-course JIA persisting into adulthood, RF positivity was associated with higher erosion and total Sharp-van der Heijde scores, while ACPA positivity was associated with higher erosion and total scores after adjustment for disease duration (4). However, the small, retrospectively selected adult cohort provides Limited evidence for predicting structural outcomes from childhood onset. A cross-disease analysis of registration trials for infliximab, tocilizumab, golimumab, and adalimumab found that exposure at the polyarticular-course JIA trial doses was similar to or higher than that in adult RA for all evaluated agents except infliximab in children weighing 35 kg or less; most comparable ACR response components were also similar to or higher than those observed in adult RA (19). This analysis provides Supportive evidence for efficacy extrapolation when pediatric exposure is adequately matched, but it included the broader polyarticular-course JIA population, was not stratified by RF status, and did not establish RF-positive-specific drug sequencing, comparative effectiveness, or long-term pediatric safety. Adult RA evidence therefore has Conditional transferability to RF-positive polyarticular JIA at the level of shared therapeutic targets and selected efficacy extrapolation, but it does not establish complete treatment equivalence.

For polyarthritis, the 2026 ACR guideline summary no longer assumes a uniform csDMARD-first sequence. It strongly recommends a csDMARD and/or bDMARD as first-line disease-modifying therapy; when a csDMARD is selected, methotrexate is conditionally preferred, whereas a TNF inhibitor is conditionally preferred when a first bDMARD is selected. Although the first-line DMARD recommendation is strong, its supporting evidence is rated very low, and the preferences for methotrexate and TNF inhibition are conditional (89). Guidance after failure of a first TNF inhibitor has also broadened. The 2019 ACR guideline conditionally preferred switching to a non-TNF biologic such as tocilizumab or abatacept over switching to a second TNF inhibitor, while allowing a second TNF inhibitor after an initial response followed by secondary failure (96). By contrast, the 2026 summary allows dose escalation of the first TNF inhibitor, switching to a second TNF inhibitor, or changing to another mechanism without identifying a preferred strategy (89). JAK inhibitors provide additional targeted options. In a phase 3 randomized withdrawal trial, disease flare occurred in 29% of 72 patients continuing tofacitinib and 53% of 70 patients switched to placebo (hazard ratio 0.46, 95% CI 0.27–0.79) (97). In a separate phase 3 randomized withdrawal trial, 163 initial responders were randomized, and continuation of baricitinib reduced the risk of flare relative to placebo (hazard ratio 0.241, 95% CI 0.128–0.453) (98). Neither trial was restricted to RF-positive polyarticular JIA, and both used responder-enriched withdrawal designs; they therefore provide Supportive evidence for maintenance efficacy in the enrolled populations but only Limited evidence for RF-positive-specific efficacy, comparative positioning against bDMARDs, or long-term safety (97, 98). When methotrexate is selected as the initial csDMARD, its early use and subsequent mechanism-based escalation parallel adult RA practice (87), but treatment transferability remains Conditional because pediatric sequencing must follow direct pediatric evidence and long-term safety remains incompletely characterized (89, 92).

### 8.4 Oligoarticular JIA: local treatment and uveitis-directed therapy

Oligoarticular JIA is managed according to limited joint burden, disease activity, and pediatric risk factors for poor outcome rather than an adult RA-like systemic step-up sequence. The 2026 ACR guideline summary conditionally recommends a trial of scheduled oral NSAIDs and strongly recommends intra-articular glucocorticoids as first-line therapy, while conditionally recommending against oral glucocorticoids as adjuncts to initial therapy. If the response to NSAIDs and/or intra-articular glucocorticoids is inadequate, a csDMARD is strongly recommended; methotrexate is conditionally preferred over leflunomide, sulfasalazine, or hydroxychloroquine, and oral administration is conditionally preferred over subcutaneous administration. A bDMARD is strongly recommended after an inadequate response to or intolerance of NSAIDs and/or intra-articular glucocorticoids and a first-line csDMARD, with a TNF inhibitor conditionally preferred as the first bDMARD over IL-6 inhibition, T-cell costimulation modulation, IL-17 inhibition, or IL-12/23 inhibition. Although several recommendations are strong, their supporting evidence ranges from low to very low certainty (89).

Uveitis requires a separate ocular risk-stratification, screening, and treatment pathway. ANA positivity contributes to risk assessment but is not a therapeutic target; screening frequency should be based on JIA category, age at disease onset, ANA status, and disease duration, with ophthalmic screening every three months for children at high risk (89, 99). For active chronic anterior uveitis, adding or increasing topical prednisolone acetate 1% is conditionally recommended for short-term control rather than adding systemic glucocorticoids. When systemic therapy is required, methotrexate is conditionally preferred over leflunomide, mycophenolate, or cyclosporine, and oral methotrexate is conditionally preferred over subcutaneous administration; adalimumab is conditionally preferred among the evaluated biologic and targeted agents (89). The oral-route recommendation differs from the 2019 ACR guideline, which conditionally preferred subcutaneous methotrexate. Because both route recommendations are based on very-low-certainty evidence, tolerability, adherence, and patient preference should guide the route of administration (89, 99).

Direct randomized evidence supports adding adalimumab when uveitis remains active despite methotrexate. In the SYCAMORE trial, the prespecified stopping boundary was met after 90 of the planned 114 participants with active JIA-associated uveitis despite stable methotrexate therapy had been randomized in a 2:1 ratio to adalimumab or placebo; treatment failure occurred in 27% and 60%, respectively (hazard ratio 0.25, 95% CI 0.12–0.49) (100). Adverse-event and serious-adverse-event rates were higher with adalimumab than with placebo (100). This randomized trial provides Supportive evidence for adding adalimumab in methotrexate-refractory JIA-associated uveitis, but early termination and the small sample limit precision and long-term safety inference. The findings do not support treatment of ANA positivity alone or routine biologic treatment of all patients with oligoarticular JIA. Adult RA principles of timely DMARD escalation have Conditional transferability when arthritis persists or extends (87), whereas the local injection-first strategy and uveitis pathway have Low transferability because they are specific to pediatric disease phenotypes.

### 8.5 Enthesitis-related arthritis and juvenile psoriatic arthritis: TNF- and IL-17-directed strategies

Enthesitis-related arthritis (ERA) belongs to the juvenile spondyloarthritis spectrum, whereas juvenile psoriatic arthritis (JPsA) is heterogeneous: some children show an early-onset ANA-positive oligoarticular phenotype, whereas others show a later-onset enthesitis, spondyloarthritis, or psoriatic-spectrum phenotype (8, 23). Treatment should therefore be matched to the dominant clinical domain—including peripheral arthritis, enthesitis, sacroiliitis or axial inflammation, dactylitis, and psoriasis—rather than follow an adult RA-like synovitis pathway. For active enthesitis, the 2026 ACR guideline summary conditionally recommends scheduled oral NSAIDs as adjunctive therapy and strongly recommends a csDMARD and/or bDMARD as first-line disease-modifying therapy; methotrexate is conditionally preferred over sulfasalazine when a csDMARD is selected, whereas a TNF inhibitor is conditionally preferred over IL-17 or IL-12/23 inhibition when a first bDMARD is selected (89). For active sacroiliitis that persists despite NSAID therapy, the 2019 ACR guideline strongly recommends adding a TNF inhibitor and strongly recommends against methotrexate monotherapy, although methotrexate may be used adjunctively when peripheral polyarthritis coexists (96). For dactylitis, the 2026 summary strongly recommends intra-articular glucocorticoid injections and tendon injections as adjunctive measures and DMARDs as first-line disease-modifying therapy; methotrexate and TNF inhibition are conditionally preferred as the first csDMARD and bDMARD options, respectively (89). Although several recommendations are strong, the supporting evidence is predominantly of low or very low certainty (89, 96).

Secukinumab provides direct pediatric evidence for IL-17A inhibition in ERA and JPsA. In a phase 3 randomized withdrawal trial, 86 biologic-naïve patients aged 2 to <18 years with active disease and an inadequate response to conventional therapy received open-label secukinumab; 75 JIA-ACR30 responders were subsequently randomized to continue secukinumab or receive placebo. Disease flare occurred in 27% and 55%, respectively (hazard ratio 0.28, 95% CI 0.13–0.63; p<0.001) (101). Category-specific estimates were less certain: the hazard ratio was 0.45 (95% CI 0.16–1.28; p=0.075) in ERA and 0.15 (95% CI 0.04–0.57; p<0.001) in JPsA, but the small subgroup sizes preclude firm comparisons between the two phenotypes (101). The trial randomized only initial responders, did not compare secukinumab directly with a TNF inhibitor, included a small and predominantly White population, and did not evaluate psoriatic skin outcomes in JPsA; rare or delayed adverse outcomes also could not be adequately characterized. It therefore provides Supportive evidence for maintenance efficacy across the combined population but only Limited evidence for subtype-specific drug sequencing. The general principle of timely treatment escalation has Conditional transferability from adult RA, whereas RA-specific csDMARD and biologic sequencing has Low transferability when enthesitis, axial inflammation, dactylitis, or psoriasis predominates. Adult spondyloarthritis and psoriatic arthritis provide the more relevant adult comparators (8, 23).

### 8.6 Target-informed treatment and current limits of molecular stratification

Current JIA treatment is phenotype- and target-informed but not biomarker-guided. The dominant therapeutic target is defined by the inflammatory compartment—including systemic hyperinflammation and MAS, peripheral synovitis, enthesitis or axial disease, dactylitis and psoriatic manifestations, or uveitis—together with disease activity, prognostic features, previous treatment exposure, comorbidities, and safety. Conventional biomarkers support classification, disease monitoring, or complication-risk assessment, but none has demonstrated prospective clinical utility for selecting one JIA therapy over another. Evidence for baseline S100–response associations under abatacept is Limited (75), pretreatment metabolomic prediction of methotrexate non-response is Exploratory (86), and baricitinib-associated serum-protein changes are pharmacodynamic associations rather than validated pretreatment predictors (85). These treatment-specific and platform-dependent findings derive from *post hoc* or small single-center analyses, often include heterogeneous JIA phenotypes, and lack standardized clinical cut-offs, independent prospective validation, and evidence of longitudinal stability; cost and assay accessibility may further restrict implementation.

Adult RA has progressed further toward treatment-specific molecular stratification: pretreatment synovial RNA models have undergone independent validation for selected therapies, and a blood-based molecular classifier has been evaluated prospectively in clinical care (72, 84). However, the classifier-informed study used propensity-matched external comparators rather than randomized biomarker-guided assignment, and neither approach supports universal routine applicability. The methodological principles of predefined clinical purpose, treatment-specific validation, and prospective clinical-utility testing have Conditional transferability to JIA, whereas direct transferability of adult RA-derived molecular signatures is Low because pediatric phenotypes, tissues, developmental stages, dosing, and treatment pathways differ. Treat-to-target assessment and timely escalation likewise have Conditional transferability from adult RA (87, 88), but individual agent selection in JIA must remain phenotype-specific and account for direct pediatric efficacy and safety evidence, age, regional regulatory availability, comorbidities, and the affected tissue compartment (6, 89, 96). Prospective pediatric trials demonstrating that biomarker-guided treatment improves outcomes over phenotype-based care are required before molecular stratification can guide routine prescribing (**Figure 3**; **Table 3**).

**Figure 3:**
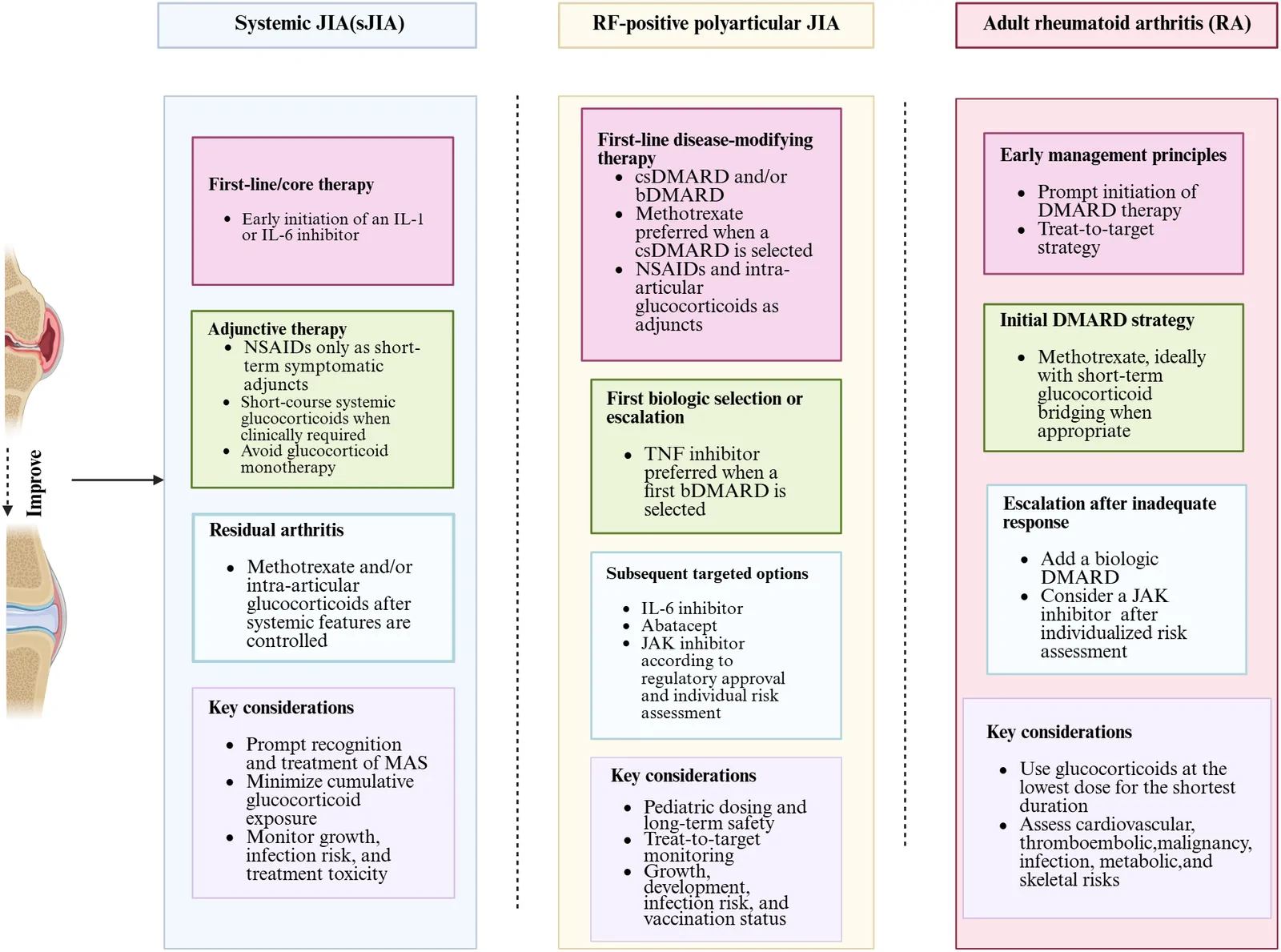
Comparative treatment strategies for systemic juvenile idiopathic arthritis, rheumatoid factor-positive polyarticular juvenile idiopathic arthritis, and adult rheumatoid arthritis. Systemic JIA requires early cytokine-directed therapy, whereas RF-positive polyarticular JIA and adult RA share methotrexate-based and mechanism-directed treatment approaches. Adult RA treatment principles have Conditional transferability to RF-positive polyarticular JIA but Low transferability to cytokine-dominant systemic JIA. In adult RA, JAK inhibitor use requires individualized risk assessment; pediatric treatment sequencing, dosing, and long-term safety require direct pediatric evidence.

**Table 3:** Domain-specific transferability of evidence derived from adult rheumatoid arthritis across representative juvenile idiopathic arthritis phenotypes.

Disease phenotype	Endotypic proximity and developmental compatibility	Immune pathway and tissue compartment	Structural consequences	Biomarker transferability	Treatment transferability	Evidence strength and principal limitations
Systemic JIA (within the Still’s disease spectrum)	Low relative to adult RA during the systemic/MAS phase; Conditional during persistent afebrile arthritis.	Low for transfer of the adult RA model to the systemic/MAS phase dominated by IL-1, IL-6, IL-18, and IFN-γ pathways; Conditional for persistent synovitis.	Conditional for inflammation-related joint damage; Low for growth-plate outcomes and systemic complications.	Low: the RA autoantibody framework does not transfer; ferritin, S100 proteins, and IL-18 are phenotype-specific.	Low for active systemic/MAS disease, in which early IL-1- or IL-6-directed therapy is prioritized; Conditional for residual arthritis after systemic control.	Early innate cytokine dominance is Established; the proposed transition toward adaptive immune-driven persistent arthritis is Supportive; treatment-response and prospective MAS-prediction biomarkers remain Limited.
RF-positive polyarticular JIA	High for the genetic and autoantibody phenotype; Conditional because growth and puberty modify expression.	High for the broad autoantibody-associated adaptive-immune model; Conditional for specific adult synovial immune–stromal mechanisms.	Conditional for erosive risk; Low for outcomes dependent on growth-plate or pubertal biology.	High for phenotype recognition; Conditional for structural prognosis; Low for treatment selection.	Conditional: MTX and bDMARD strategies overlap with adult RA, whereas JAK inhibitors require pediatric efficacy, dosing, and long-term safety evidence; treatment sequencing remains pediatric-specific.	Genetic overlap is Supportive; structural prognosis is Limited; treatment trials include mixed JIA phenotypes.
Oligoarticular JIA	Low: local pediatric synovitis and uveitis lack a close adult RA endotype.	Low for the RA autoantibody model; Conditional for methodological transfer of tissue-profiling approaches.	Conditional for generic synovitis-related damage; Low for growth disturbance and uveitis.	Low for transfer from adult RA; the ANA–uveitis association is Established, but individualized prediction is Limited.	Conditional for persistent or extended arthritis; Low for local injection-first and uveitis pathways.	T-cell mechanisms are Supportive; tissue biomarkers are Exploratory; guideline certainty is generally low.
Enthesitis-related arthritis	Low relative to adult RA; Conditional relative to adult spondyloarthritis, with developmental modifiers.	Low for RA pathways; Conditional for entheseal/axial IL-17 and mucosal mechanisms.	Low for the RA erosion model when enthesis- or axial-dominant disease predominates.	Low for transfer from adult RA; HLA-B27 has an Established classification role, while prognostic and emerging markers remain Supportive or Exploratory.	Low for RA-specific sequencing; Conditional for timely escalation; TNF- and IL-17-directed evidence is pediatric-specific.	Pediatric mechanisms are Limited or Exploratory; guideline certainty is low; secukinumab maintenance evidence is Supportive.
Juvenile psoriatic arthritis	Low relative to adult RA; Conditional relative to adult psoriatic arthritis; age-related heterogeneity is substantial.	Low for RA pathways; Conditional for adult skin–joint and IL-23/IL-17 mechanisms.	Conditional for peripheral joint damage; Low for psoriatic, dactylitic, and axial domains.	Low for transfer from adult RA: no RA-derived diagnostic or treatment-selection biomarker is validated for this phenotype.	Low for RA-specific sequencing; Conditional for treat-to-target principles; direct pediatric evidence is required.	Pediatric mechanistic evidence is Limited; the combined secukinumab trial is Supportive but lacked skin outcomes.
Adult RA reference comparator	Reference comparator after skeletal maturity; pediatric developmental compatibility is not assumed.	Established autoantibody-associated adaptive immunity and synovial immune–stromal–osteoclast networks.	Established cartilage loss and bone erosion after physeal closure.	RF and ACPA are Established; molecular treatment prediction is Supportive but not routine.	Treat-to-target and MTX-to-bDMARD escalation are Established in adult RA; JAK inhibitors may also be considered after individualized assessment of the risks of major adverse cardiovascular events, malignancy, and thromboembolic events. Pediatric transfer remains domain-specific.	Adult evidence is mature, but it is indirect for children and does not capture growth or pubertal biology.

ACPA, anti-citrullinated protein antibodies; ANA, antinuclear antibodies; bDMARD, biologic disease-modifying antirheumatic drug; HLA-B27, human leukocyte antigen B27; IFN-γ, interferon gamma; IL, interleukin; JAK, Janus kinase; JIA, juvenile idiopathic arthritis; MAS, macrophage activation syndrome; MTX, methotrexate; RA, rheumatoid arthritis; RF, rheumatoid factor; S100, S100 calcium-binding protein family; TNF, tumor necrosis factor.

Unless otherwise specified, transferability ratings refer to evidence derived from adult RA. High indicates direct concordance supported by pediatric evidence; Conditional indicates biological overlap modified by development, phenotype, tissue compartment, treatment context, or limited pediatric validation; Low indicates a substantially different dominant endotype or tissue compartment. Evidence levels (Established, Supportive, Limited, and Exploratory) describe evidence maturity and are not equivalent to transferability ratings. References to adult spondyloarthritis or adult psoriatic arthritis identify explicit phenotype-specific comparators.

## 9 Knowledge gaps and future research priorities

Current evidence supports a phenotype- and domain-specific rather than universal relationship between JIA and adult RA. RF-positive polyarticular JIA shows the strongest convergence with adult seropositive RA, whereas systemic JIA, oligoarticular JIA, enthesitis-related arthritis, and juvenile psoriatic arthritis retain distinct immune programs, tissue compartments, complications, and treatment pathways. Cross-age inference remains limited by small and subtype-heterogeneous pediatric cohorts, scarce age-stratified and tissue-based data, inconsistent biomarker validation, and insufficient longitudinal follow-up through growth, pubertal development, and prolonged treatment exposure.

Research priorities should therefore be phenotype-specific. In systemic JIA, prospective studies should evaluate biomarker combinations for predicting MAS and distinguishing cytokine-dominant systemic disease from persistent afebrile arthritis. RF-positive polyarticular JIA requires direct pediatric synovial studies and long-term imaging cohorts to determine which adult RA immune–stromal mechanisms, structural prognostic markers, and treatment-response signatures are genuinely shared. Oligoarticular JIA needs better predictors of disease extension and uveitis course, whereas enthesitis-related arthritis and juvenile psoriatic arthritis require phenotype-stratified comparison with adult spondyloarthritis and psoriatic arthritis rather than a universal RA comparator. Across phenotypes, candidate biomarkers require prespecified clinical functions, standardized assays and cut-offs, independent multicenter validation, and prospective evidence of clinical utility. Harmonized pediatric registries and biobanks linking longitudinal clinical, imaging, tissue, and treatment-safety data across pediatric and adult care would support this work. Additional priorities include translating single-cell and spatial-tissue signatures into reproducible clinical assays, integrating genetic, immune, microbiome, environmental, and treatment-exposure data, and following subtype-defined pediatric cohorts through transition to adult care. Prospective biomarker-guided trials are needed to determine whether these approaches improve outcomes beyond phenotype-based treatment.

Taken together, JIA should neither be conceptualized as a pediatric form of adult RA nor regarded as biologically unrelated to it. The most defensible approach is domain-specific, developmentally conditioned transferability: adult RA evidence is most informative for RF-positive polyarticular JIA and RA-like persistent synovitis but less applicable when systemic hyperinflammation, MAS, uveitis, enthesitis, axial disease, or psoriatic pathways predominate. Until validated pediatric molecular classifiers and treatment-selection biomarkers are available, clinical phenotype, disease activity, affected tissue compartment, developmental stage, and direct pediatric evidence should remain the basis for monitoring and treatment decisions.

## Glossary

AC-30: Fine speckled with mitotic plate antinuclear antibody pattern
ACPA: Anti-citrullinated protein antibodies
ACR: American College of Rheumatology
ANA: Antinuclear antibodies
anti-CCP: Anti-cyclic citrullinated peptide antibodies
bDMARD: Biologic disease-modifying antirheumatic drug
CCL5: C-C motif chemokine ligand 5
CD: Cluster of differentiation
CDAI: Clinical Disease Activity Index
CRP: C-reactive protein
csDMARD: Conventional synthetic disease-modifying antirheumatic drug
DMARD: Disease-modifying antirheumatic drug
ERA: Enthesitis-related arthritis
ESR: Erythrocyte sedimentation rate
EULAR: European Alliance of Associations for Rheumatology
FCAR: Fc alpha receptor (FcαRI/CD89)
GZMA: Granzyme A
HLA: Human leukocyte antigen
HLA-B27: Human leukocyte antigen B27
IFN-γ: Interferon gamma
IgA: Immunoglobulin A
IgM: Immunoglobulin M
IL: Interleukin
IL-1: Interleukin-1
IL-1β: Interleukin-1 beta
IL-6: Interleukin-6
IL-17: Interleukin-17
IL-18: Interleukin-18
IL-23: Interleukin-23
IL-34: Interleukin-34
JAK: Janus kinase
JAK-STAT: Janus kinase-signal transducer and activator of transcription
JIA: Juvenile idiopathic arthritis
MAS: Macrophage activation syndrome
MMP-3: Matrix metalloproteinase-3
MRI: Magnetic resonance imaging
MRP8/14: Myeloid-related proteins 8 and 14 complex
MSRC: Molecular signature response classifier
NETs: Neutrophil extracellular traps
NSAIDs: Nonsteroidal anti-inflammatory drugs
PD-1: Programmed cell death protein 1
RA: Rheumatoid arthritis
RANKL: Receptor activator of nuclear factor kappa B ligand
RF: Rheumatoid factor
RNA: Ribonucleic acid
S100A8/A9: S100 calcium-binding proteins A8 and A9 (calprotectin)
S100A12: S100 calcium-binding protein A12
SI: Sacroiliac joint
sJIA: Systemic juvenile idiopathic arthritis
Th1: T helper 1 cell
TMJ: Temporomandibular joint
TNF: Tumor necrosis factor
TNF-α: Tumor necrosis factor alpha
TNFi: Tumor necrosis factor inhibitor
tsDMARD: Targeted synthetic disease-modifying antirheumatic drug.
