# Supplementary material for: Juvenile idiopathic arthritis and rheumatoid arthritis: a narrative review of age-dependent, subtype-specific immune mechanisms, biomarkers, and treatment strategies
Source: Front Immunol. 2026 Oct 27;17:1873473. doi: 10.3389/fimmu.2026.1873473 (PMC13749398; doi:10.3389/fimmu.2026.1873473)
Supplement: Supplementary file 1 [file Table1.docx]

**Supplementary Table S1.**

Structured search period: 1 January 2015 to 25 September 2026; language: English*.*

| **Database** | **Search ID** | **Purpose** | **Exact search strategy** | **Records** |
| --- | --- | --- | --- | --- |
| PubMed/MEDLINE | P1 | Direct comparison of JIA with adult RA | (("Arthritis, Juvenile"[Mesh] OR "juvenile idiopathic arthritis"[tiab] OR "juvenile rheumatoid arthritis"[tiab] OR "systemic juvenile idiopathic arthritis"[tiab] OR "systemic JIA"[tiab] OR "polyarticular juvenile idiopathic arthritis"[tiab] OR "polyarticular JIA"[tiab] OR "RF-positive polyarticular JIA"[tiab] OR "oligoarticular juvenile idiopathic arthritis"[tiab] OR "oligoarticular JIA"[tiab] OR "enthesitis-related arthritis"[tiab] OR "juvenile psoriatic arthritis"[tiab]) AND ("Arthritis, Rheumatoid"[Mesh] OR "rheumatoid arthritis"[tiab] OR "adult rheumatoid arthritis"[tiab] OR "seropositive rheumatoid arthritis"[tiab])) AND english[la] AND ("2015/01/01"[dp] : "2026/09/25"[dp]) | 930 |
| PubMed/MEDLINE | P2 | Phenotype- and domain-specific JIA evidence | (("juvenile idiopathic arthritis"[ti] OR "juvenile rheumatoid arthritis"[ti] OR "systemic juvenile idiopathic arthritis"[ti] OR "systemic JIA"[ti] OR "polyarticular juvenile idiopathic arthritis"[ti] OR "polyarticular JIA"[ti] OR "RF-positive polyarticular JIA"[ti] OR "oligoarticular juvenile idiopathic arthritis"[ti] OR "oligoarticular JIA"[ti] OR "enthesitis-related arthritis"[ti] OR "juvenile psoriatic arthritis"[ti]) AND ("age-dependent"[ti] OR developmental[ti] OR growth[ti] OR puberty[ti] OR pubertal[ti] OR "growth plate"[ti] OR skeletal[ti] OR bone[ti] OR cartilage[ti] OR muscle[ti] OR sarcopenia[ti] OR temporomandibular[ti] OR mandibular[ti] OR uveitis[ti] OR immun*[ti] OR cytokine*[ti] OR synovi*[ti] OR fibroblast*[ti] OR macrophage*[ti] OR monocyte*[ti] OR neutrophil*[ti] OR "T cell"[ti] OR "T cells"[ti] OR "B cell"[ti] OR "B cells"[ti] OR "single-cell"[ti] OR "single cell"[ti] OR "spatial transcriptomic"[ti] OR endotype*[ti] OR biomarker*[ti] OR "rheumatoid factor"[ti] OR ACPA[ti] OR "anti-CCP"[ti] OR "antinuclear antibody"[ti] OR ANA[ti] OR HLA-B27[ti] OR ferritin[ti] OR IL-18[ti] OR interleukin-18[ti] OR S100A8[ti] OR S100A9[ti] OR S100A12[ti] OR calprotectin[ti] OR microRNA*[ti] OR transcriptom*[ti] OR proteom*[ti] OR metabolom*[ti] OR treatment[ti] OR therapy[ti] OR guideline*[ti] OR "treat-to-target"[ti] OR methotrexate[ti] OR glucocorticoid*[ti] OR DMARD*[ti] OR biologic*[ti] OR "TNF inhibitor"[ti] OR "TNF inhibitors"[ti] OR "IL-1 inhibitor"[ti] OR "IL-1 inhibitors"[ti] OR "IL-6 inhibitor"[ti] OR "IL-6 inhibitors"[ti] OR "IL-17 inhibitor"[ti] OR "IL-17 inhibitors"[ti] OR "JAK inhibitor"[ti] OR "JAK inhibitors"[ti] OR anakinra[ti] OR canakinumab[ti] OR tocilizumab[ti] OR abatacept[ti] OR adalimumab[ti] OR etanercept[ti] OR tofacitinib[ti] OR baricitinib[ti] OR secukinumab[ti] OR safety[ti])) AND english[la] AND ("2015/01/01"[dp] : "2026/09/25"[dp]) | 1,746 |
| PubMed/MEDLINE | P3 | Adult RA comparator reviews and guidelines | ("rheumatoid arthritis"[ti] AND (pathogenesis[ti] OR mechanism*[ti] OR synovi*[ti] OR biomarker*[ti] OR "treatment response"[ti] OR "targeted therapy"[ti] OR "treat-to-target"[ti] OR management[ti] OR guideline*[ti]) AND (systematic review[pt] OR meta-analysis[pt] OR review[pt] OR practice guideline[pt] OR guideline[pt])) AND english[la] AND ("2015/01/01"[dp] : "2026/09/25"[dp]) | 932 |
| PubMed/MEDLINE | P4 | Unique PubMed set | P1 OR P2 OR P3 | 3,509 |
| Web of Science Core Collection (SCI-EXPANDED) | W1 | Direct JIA-RA comparison | TS=("juvenile idiopathic arthritis" OR "juvenile rheumatoid arthritis" OR "systemic juvenile idiopathic arthritis" OR "systemic JIA" OR "polyarticular juvenile idiopathic arthritis" OR "polyarticular JIA" OR "RF-positive polyarticular JIA" OR "oligoarticular juvenile idiopathic arthritis" OR "oligoarticular JIA" OR "enthesitis-related arthritis" OR "juvenile psoriatic arthritis") AND TS=("rheumatoid arthritis" OR "adult rheumatoid arthritis" OR "seropositive rheumatoid arthritis") AND PY=(2015-2026) AND LA=(English) | 2,105 |
| Web of Science Core Collection (SCI-EXPANDED) | W2 | Phenotype- and domain-specific JIA evidence | TI=("juvenile idiopathic arthritis" OR "juvenile rheumatoid arthritis" OR "systemic juvenile idiopathic arthritis" OR "systemic JIA" OR "polyarticular juvenile idiopathic arthritis" OR "polyarticular JIA" OR "RF-positive polyarticular JIA" OR "oligoarticular juvenile idiopathic arthritis" OR "oligoarticular JIA" OR "enthesitis-related arthritis" OR "juvenile psoriatic arthritis") AND TI=("age-dependent" OR developmental OR growth OR puberty OR pubertal OR "growth plate" OR skeletal OR bone OR cartilage OR muscle OR sarcopenia OR temporomandibular OR mandibular OR uveitis OR immun* OR cytokine* OR synovi* OR fibroblast* OR macrophage* OR monocyte* OR neutrophil* OR "T cell" OR "T cells" OR "B cell" OR "B cells" OR "single-cell" OR "single cell" OR "spatial transcriptomic" OR endotype* OR biomarker* OR "rheumatoid factor" OR ACPA OR "anti-CCP" OR "antinuclear antibody" OR ANA OR HLA-B27 OR ferritin OR IL-18 OR interleukin-18 OR S100A8 OR S100A9 OR S100A12 OR calprotectin OR microRNA* OR transcriptom* OR proteom* OR metabolom* OR treatment OR therapy OR guideline* OR "treat-to-target" OR methotrexate OR glucocorticoid* OR DMARD* OR biologic* OR "TNF inhibitor" OR "TNF inhibitors" OR "IL-1 inhibitor" OR "IL-1 inhibitors" OR "IL-6 inhibitor" OR "IL-6 inhibitors" OR "IL-17 inhibitor" OR "IL-17 inhibitors" OR "JAK inhibitor" OR "JAK inhibitors" OR anakinra OR canakinumab OR tocilizumab OR abatacept OR adalimumab OR etanercept OR tofacitinib OR baricitinib OR secukinumab OR safety) AND PY=(2015-2026) AND LA=(English) | 2,640 |
| Web of Science Core Collection (SCI-EXPANDED) | W3 | Adult RA comparator reviews and recommendations | TI=("rheumatoid arthritis") AND ((TI=(pathogenesis OR mechanism* OR synovi* OR biomarker* OR "treatment response" OR "targeted therapy" OR "treat-to-target" OR management) AND DT=(Review)) OR TI=(guideline* OR recommendation* OR consensus*)) AND PY=(2015-2026) AND LA=(English) | 961 |
| Web of Science Core Collection (SCI-EXPANDED) | W4 | Combined Web of Science set | #1 OR #2 OR #3 | 5,207 |

**Web of Science settings:** Core Collection; SCI-EXPANDED (2008-present); timespan 2015–2026; English. W1–W3 were run consecutively and combined as #1 OR #2 OR #3; W4 was exported for deduplication.

**Study-selection counts:** The structured database searches identified 3,509 unique PubMed/MEDLINE records and 5,207 Web of Science records, yielding 8,716 database records before cross-database deduplication. An additional 47 records were identified through reference-list screening and supplementary sources. After 2,777 duplicates were removed, 5,986 records remained for title-and-abstract screening. The final narrative synthesis included 117 unique publications. The number of full-text reports assessed was not separately documented because full-text assessment was not prospectively recorded as a distinct PRISMA-style stage.

**Note:** Earlier landmark studies outside the structured 2015–2026 search window were identified through reference-list screening and were included among the 47 additional records when they established classification systems, foundational mechanisms, validated outcome measures, or treatment principles.
